# Bioinformatics analysis of thousands of TCGA tumors to determine the involvement of epigenetic regulators in human cancer

**DOI:** 10.1186/1471-2164-16-S8-S5

**Published:** 2015-06-18

**Authors:** Florian Gnad, Sophia Doll, Gerard Manning, David Arnott, Zemin Zhang

**Affiliations:** 1 Departments of Bioinformatics and Computational Biology, Genentech, USA; 2 Protein Chemistry, Genentech Inc., South San Francisco, CA 94080, USA; 3 Proteomics and Signal Transduction, Max-Planck-Institute for Biochemistry, D-82152 Martinsried, Germany; 4 Biodynamic Optical Imaging Center, College of Life Sciences, Peking University, Beijing 100871, China

## Abstract

**Background:**

Many cancer cells show distorted epigenetic landscapes. The Cancer Genome Atlas (TCGA) project profiles thousands of tumors, allowing the discovery of somatic alterations in the epigenetic machinery and the identification of potential cancer drivers among members of epigenetic protein families.

**Methods:**

We integrated mutation, expression, and copy number data from 5943 tumors from 13 cancer types to train a classification model that predicts the likelihood of being an oncogene (OG), tumor suppressor (TSG) or neutral gene (NG). We applied this predictor to epigenetic regulator genes (ERGs), and used differential expression and correlation network analysis to identify dysregulated ERGs along with co-expressed cancer genes. Furthermore, we quantified global proteomic changes by mass spectrometry after EZH2 inhibition.

**Results:**

Mutation-based classifiers uncovered the OG-like profile of *DNMT3A *and TSG-like profiles for several ERGs. Differential gene expression and correlation network analyses revealed that *EZH2 *is the most significantly over-expressed ERG in cancer and is co-regulated with a cell cycle network. Proteomic analysis showed that EZH2 inhibition induced down-regulation of cell cycle regulators in lymphoma cells.

**Conclusions:**

Using classical driver genes to train an OG/TSG predictor, we determined the most predictive features at the gene level. Our predictor uncovered one OG and several TSGs among ERGs. Expression analyses elucidated multiple dysregulated ERGs including *EZH2 *as member of a co-expressed cell cycle network.

## Background

The epigenetic landscape has become an important research topic within oncology. Epigenetic regulatory mechanisms include DNA methylation, covalent histone modification, and chromatin remodeling mediated by the SWI/SNF complex. DNA methylation typically reduces gene expression and is catalyzed by three major DNA methyltransferases (DNMTs) [1]. In comparison, a larger and more diverse panel of proteins regulates gene expression as writers, readers or erasers of posttranslational histone modifications [2,3] (Additional file 1). Acetyl marks are written by histone acetyltransferases (HATs), read by bromodomain containing proteins, and erased by histone deacetylases (HDACs). Analogously, histone methyl marks are written by methyltransferases (HMTs) and erased by demethylases (HDMTs). The multi-subunit SWI/SNF chromatin-remodeling complex modulates gene expression via nucleosome repositioning [4].

Perturbing the epigenetic machinery can lead to uncontrolled cellular proliferation and altered apoptosis [5,6]. Consequently, alterations of epigenetic regulators and histone marks are frequently observed in cancer and numerous compounds have been reported to be effective against cancer cells by inhibiting epigenetic proteins and reversing the effect of epigenetic modifications [7,8]. Clinically approved epigenetic drugs include the DNMT inhibitors vidaza and decitabine [9,10], and HDAC inhibitors vorinostat and romidepsin [11,12] for treatment of myelodysplastic syndrome and cutaneous T cell lymphoma, respectively. Multiple pharmaceutical companies are targeting the histone methyltransferase EZH2 for cancer treatment. EZH2 inhibitors EPZ-5687 [13] from Epizyme^®^ and GSK-2816126 [14] from GlaxoSmithKline^®^ for the treatment of non-Hodgkin's lymphoma are currently in clinical phases I/II and I, respectively.

The increasing interest in the role of epigenetic mechanisms in cancer has been accompanied by technological breakthroughs and large-scale initiatives to profile large numbers of human tumors. The TCGA network has produced genome and transcriptome sequencing data for thousands of tumors, allowing systematic analysis of molecular defects in cancer [15-17]. Integrative analyses as seen in the TCGA Pan-Cancer project [18] can uncover OGs and TSGs, identify novel biomarkers, and classify molecular subtypes. Most of the current driver identification approaches aim to uncover somatic alterations, point mutations in particular, that occur at a statistically significant rate in cancer. Alternatively, using genomic profiles of known OGs and TSGs as a reference, machine learning based predictors can be trained to identify cancer genes [19].

Both methodologies are founded on features of classical drivers, which are mainly characterized by significant mutation or copy number patterns. Many other genes show consistent deregulated expression in cancer, but are not classified as drivers, because their impact on the development of cancer is not clear. Mutation based analyses might therefore underestimate the roles of genes that drive cancer via increased or decreased expression. Here we analyze the genomic landscapes of thousands of tumors to pinpoint molecular aberrations within ERG families. We used mutation, expression, and copy number alterations as features to predict OGs and TSGs among 187 epigenetic regulators based on both published and reprocessed TCGA data. Differential gene expression analysis revealed ERGs with frequent distorted expression in cancer. We further aimed to identify genes that are co-expressed with ERGs.

## Materials and methods

### Definition of ERGs and application of predictors

We classified ERG families by the presence of domains associated with writing, reading and erasing epigenetic marks, and defined the relationships between their members by sequence similarity. For deacetylases, methyltransferases, demethylases, and bromodomain-containing proteins, the amino acid sequences of the corresponding domains were used to determine conservation by multiple sequence alignment. Domain annotations were retrieved from the UniProt database (http://www.uniprot.org) [20]. Sequences of 'SET', 'SAM-dependent MTase PRMT-type' and 'DOT1' domains were derived for methyltransferases. 'JmjC' and 'SWIRM' domains were characteristic for demethylases. Deacetylases contained 'Histone deacetylase' or 'Deacetylase sirtuin-type' domains, while each bromodomain containing protein contained at least one domain described as 'Bromo' or 'Bromo 1' in UniProt. When proteins had multiple copies of a domain, the N-terminal domain was used. Full-length sequences were used for acetyltransferases and members of the SWI/SNF complex, since their catalytic domains are not clearly defined. We created multiple sequence alignments for each family with ClustalW2 (http://www.ebi.ac.uk/Tools/msa/clustalw2/) [21] using default parameters. Phylogenetic trees were calculated with Jalview 2.8 [22] based on average distance minimization and visualized in iTOL 2.1 (http://itol.embl.de) [23,24].

### Mutation and copy number data

To create gene-alteration profiles for all human genes, mutation and copy number data from tumors across the following published TCGA cancer types were retrieved using cBioPortal (http://cbioportal.org) [25,26]: urothelial bladder carcinoma (BLCA) [27], breast carcinoma (BRCA) [28], colon and rectal carcinoma (COAD, READ) [29], glioblastoma (GBM) [30], chromophobe renal cell carcinoma [31] (KICH), clear cell renal carcinoma (KIRC) [32], acute myeloid leukemia (LAML) [33], lung adenocarcinoma (LUAD) [34], lung squamous cell carcinoma (LUSC) [35], ovarian carcinoma (OV) [36], gastric adenocarcinoma (STAD) [37], papillary thyroid carcinoma (THCA) [38], and endometrial carcinoma (UCEC) [39]. The CGDSR R package functions *getMutationData *and *getProfileData *were recursively applied for all RefSeq genes. We distinguished between missense mutations with high (HiFI) or low (LoFI) functional impact based on MutationAssessor [40]. Mutations with predicted "medium" or "high" functional impacts were defined as HiFI mutations, while mutations with predicted "neutral" or "low" functional impacts were defined as LoFI mutations. Loss of function (LOF) mutations were determined as the sum of nonsense and frameshift mutations. In addition to non-synonymous mutations from cBioPortal, we retrieved silent mutations directly from the TCGA Data Portal (https://tcga-data.nci.nih.gov/tcga/). Benign mutations were defined as the combination of silent and LoFI mutations. Copy number levels from cBioPortal were classified as 'deep loss', 'single-copy loss', 'diploid', 'low-level gain' or 'high-level gain' by GISTIC [41]. The extents of copy number deletions and amplifications for each gene in each cancer study were determined as the proportions of tumors with 'deep loss' and 'high-level gain' changes, respectively. R [42] was used to format mutation and copy number data for annotation of trees in iTOL.

### Expression data and differential gene expression analysis

To identify differential gene expression between tumors and healthy tissues, TCGA RNAseq raw reads were downloaded for available tumor types (BLCA, BRCA, COAD, KICH, KIRC, LUAD, LUSC, STAD, TCHA, UCEC) and processed by our GSNAP [43] based transcriptome analysis pipeline [44]. RNAseq data for both tumors and healthy tissues were not available for GBM, LAML and OV. RNAseq reads were first aligned to ribosomal RNA sequences to remove ribosomal reads. Remaining reads were aligned to the human reference genome (NCBI Build 37) using GSNAP version '2012-01-11', allowing maximum of 2 mismatches per 75 base sequence (parameters: "-M 2 -n 10 -B 2 -i 1 -N 1 -w 200000 -E 1 --pairmax-rna = 200000"). Gene expression was quantified with RPKM values (reads mapping to a gene per kilobase of transcript per million reads sequenced) and variance stabilized counts derived from the number of reads mapped to each RefSeq gene. The DESeq R package [45] was applied to estimate size factors, obtain dispersion estimates, and measure differential gene expression between tumors and healthy tissues using default parameters. Results were reported as fold changes and associated adjusted p-values. In addition to DESeq based negative binomial generalized linear models for differential expression significance, we defined genes with tumor exclusive expression (genes that are expressed in tumors but not in healthy tissues), if their 90% quantile expression levels in all healthy tissues were equal to the expression levels of pseudo counts, but minimum 1 RPKM in the tumors of at least one cancer type.

### Prediction of OGs and TSGs

Following the methodology for parameter tuning as described in the TUSON explorer [19], we applied the Lasso approach [46] to identify the most reliable features for predicting OGs and TSGs. Lasso minimizes the residual sum of squares (RSS) with a constraint ("L1 penalty") on the sum of the absolute values of the coefficients *β_j _*for all predictors p:

$$RSS+\lambda \sum_{j=1}^{p}|\beta_{j}|$$

The L1 penalty has the effect of shrinking some of the coefficients to zero when the tuning parameter λ is sufficiently large. As a result, lasso models select the most predictive subsets of features at specified λ values.

For both feature selection and training, we used 49 OGs and 49 TSGs from the Cancer Gene Census (CGC) [47] with experimentally validated involvement in tumorigenesis as provided by TUSON. Genes that have not been associated with cancer development according to CGC or the Entrez gene database formed a set of 10,900 NGs. Using TCGA data we employed 48 features associated with mutation, expression, or copy number alterations for each human gene (Additional file 2). To prevent imbalanced classifications, we created 1000 random NG sets of size 150 each. Feature selections and predictions were conducted for OGs and TSGs separately.

Using the 'cv.glmnet' function from the R package glmnet [46], we trained lasso based binomial classification models for each random NG set against all OGs or TSGs. We used 20-fold cross validations to determine tuning parameter λ yielding minimum cross-validated errors. Features were defined as reliable for OG or TSG prediction, respectively, if the associated β coefficients were not zero in at least 90% of the 1000 resulting classifiers. While TUSON applied the lasso approach for feature selection only, we also used the resulting fitted logistic regression models for prediction. We applied glmnet's 'predict' function to each of the 1000 fitted models based on optimal λ values and the respective optimal feature subsets. This resulted into 1000 sets of predicted OGs and TSGs. Using a bagging based ensemble classification approach, we applied binary classifications of all human genes based on a 90% majority vote.

Notably, we used all 49 OGs and 49 TSGs as positive sets for training. In the absence of a separate test set, prediction accuracies were therefore measured as average 20-fold cross validation based areas under the curve (AUC) across the 1000 classifiers from the training step.

### Co-expression analysis

To estimate the strength of the pairwise linear relationship between the expression levels all human genes in healthy tissues, Pearson's correlation coefficients were calculated based on WGCNA, an R package for weighted correlation network analysis [48]. Using DESeq [45], variance stabilized RNAseq count data were used as a measure of gene expression. Expression data of all non-tumor samples were merged and analyzed in a combined approach. We applied hard thresholding (R > 0.85) to convert the resulting 19,115 × 19,115 similarity matrix into an adjacency matrix. Using R we turned the adjacency matrix into a network file that can be imported in Cytoscape [49]. Known cancer genes were defined by the Cancer Gene Census (CGC) [47]. In total 25 out of 501 CGC genes were ERGs.

### Sample preparation and mass spectrometry analysis

To analyze the effect of EZH2 inhibition on the proteome, we applied quantitative mass spectrometry based proteomics to a non-Hodgkin's lymphoma B cell line, WSU-DLCL2. Cells were cultivated in SILAC RMPI 1640 medium containing ^13^C6^15^N2-lysine (Lys8) and ^13^C6^15^N4-arginine (Arg10), as described [50]. After fully labeling, as assessed by quantitative mass spectrometry, cells were treated with the EZH2 inhibitor EPZ-6438 (Epizyme^®^, Cambridge, MA) (provided by LT PharmaTech Inc^®^) (250 nM) for 2, 4, 6 or 8 days.

Cell pellets were lysed in 8 M Urea, 20 mM HEPES buffer by sonication and clarified by centrifugation at 16,000 × g for 10 min. Protein content was measured using the Pierce BCA protein assay (Thermo Scientific) by fluorescence spectrometry. SILAC-labeled proteins were combined with an equal amount of unlabeled proteins. Proteins were reduced with dithiothreitol and alkylated with iodoacetamide prior to tryptic in-gel digestion. 100 µg of the heavy/light protein mix was loaded and separated by SDS-PAGE on a 4-12% NuPAGE Bis-Tris gel (Invitrogen) and stained with SimplyBlue Coomassie (Invitrogen). Gel bands were excised, separated into 16 fractions, and destained followed by overnight trypsin digestion at 37°C in 50 mM ammonium bicarbonate.

Nanoflow LC-MS/MS analysis of tryptic peptides was conducted on an LTQ-Orbitrap XL (ThermoFisher) in combination with a Waters nanoAcquity UPLC system, as described [50]. The mass spectrometer was operated in data-dependent mode and tandem mass spectra were searched against the UniProt human database using Mascot and a maximum false positive rate of 2% for proteins.

### Histone purification and H3K27me3 quantification

H2A, H2B, H3, and H4 histones were purified with a commercially available histone purification kit (Active Motif) accordingly to the manufacturer's instruction. Histone concentrations were measured using the Direct Detect^®^ Spectrometer (EMD Millipore). Heavy and light amino acid-labeled histones were mixed in a 1:1 ratio. Histones were propionylated, quenched by hydroxylamine followed by tryptic digestion overnight and phenyl isocyanate labeling. Histone peptides were then analyzed by capillary reverse phase ultra high-pressure liquid chromatography-electrospray ionization tandem mass spectrometry on an Orbitrap mass spectrometer. Briefly, 1 µg of desalted histone peptides were injected on 1.7 µm BEH-C18 column (Waters) and eluted over the course of 90 minutes with an acetonitrile gradient. Spectra were acquired in a "top-15" data-dependent experiment. Data were further processed with Fishtones (http://research-pub.gene.com/fishtones-js/howto/.)

### Clustering of time courses

Using the R package Mfuzz [51], log2 ratios of protein intensity time profiles were clustered based on the fuzzy c-means (FCM) soft partitioning clustering algorithm. We used c = 3 and m = 1.7 as parameters, where c is the number of clusters and m is the fuzzification parameter. Membership values ranging from 0 to 1 reflect the similarities of each time profile to its associated cluster.

### Gene ontology analysis

We used Cytoscape [49] and BinGO [52] to derive biological functions that were significantly overrepresented in co-expressed gene networks or proteins with intensity changes after EZH2 inhibition. The significance of overrepresented gene ontology annotations in these sets compared to entire human proteome was calculated on the basis of hypergeometric models and Benjamini Hochberg false discovery rate correction.

## Results

### Definition of ERG families

We defined ERG families and their members by the presence of domains associated with writing, reading and erasing epigenetic marks as described [53]. The resulting panel of 187 epigenetic regulators comprised 3 DNMTs, 58 HMTs, 32 HDMTs, 18 HATs, 18 HDACs, 41 bromodomain proteins, and 20 members of the SWI/SNF complex (Additional files 1, 3 and 4). Their phylogenetic relationships were estimated by the sequence similarity of associated domains. Using mutation, copy number and expression data from 5943 tumors across 13 TCGA cancer types, we set out to determine the involvement of the defined ERGs in human cancer by OG/TSG prediction, differential expression and correlation network analysis.

### Prediction of cancer driver genes

The most common approach to uncover cancer driver genes is to identify somatic alterations that occur at a statistically significant rate. As an alternative approach, machine learning based classifications use characteristics of known OGs and TSGs as a reference (training set) to predict cancer genes [19]. We implemented a similar approach to test its applicability in our tumor panel and to identify cancer drivers within ERG families.

#### Construction of gene-alteration profiles

**Figure 1:**
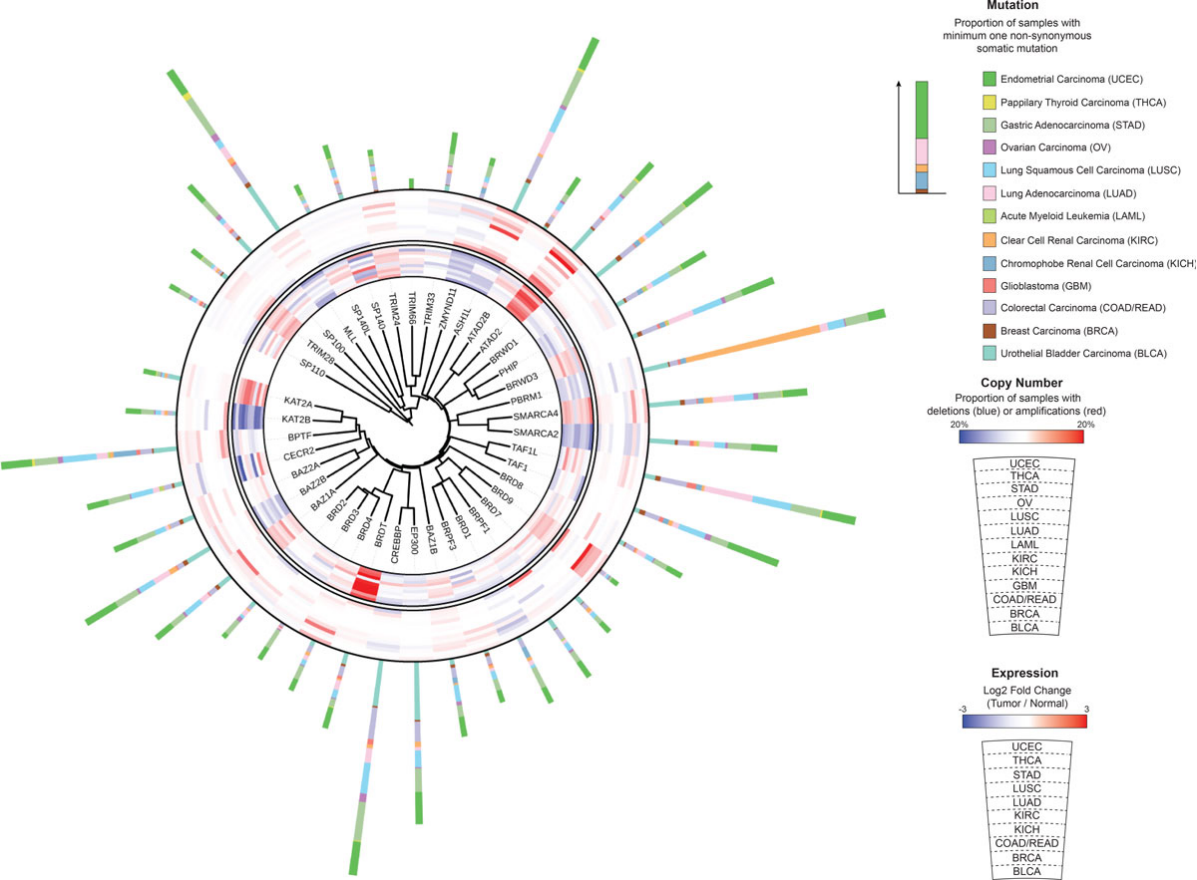
**Visualizing genomic alterations of bromo domain containing genes**. The core of the plot reflects the phylogenetic relationships between bromo domain containing proteins estimated by the sequence similarity of their associated domains. The inner circle displays the expression fold-changes between tumors and healthy tissues. High expression in tumors is indicated in red, while low expression in tumors is shown in blue. The outer circle illustrates the proportion of tumors with 'deep loss' (blue) or 'high-level gain' (red) changes. Mutation rates are reflected by the outer stacked bar plots.

To characterize known cancer drivers and to identify genes with similar features, we constructed 'alteration profiles' for all human genes in each individual cancer type as well as in the combined set of tumors (Additional file 5, Materials and Methods). Each gene profile contained 48 features measuring various types of alterations in cancer (Additional file 2). For members of the defined ERG gene families, we applied iTOL to visualize four of the 48 established features (Figure 1 and Additional file 6). These include the proportions of tumors with significant copy number alterations, non-synonymous sequence mutations within the gene coding region, and the degree of differential expression between tumors and adjacent normal tissues. The frequencies of copy number deletions or amplifications for each gene were determined as the proportions of tumors with 'deep loss' or 'high-level gain' changes based on GISTIC calculations [41], respectively. To measure the degree of dysregulated expression for each gene, we developed fold-change and p-value based scores reflecting the significance of differential gene expression based on negative binomial generalized linear models (Materials and Methods). The majority (44 of 48) of the integrated features, however, describe the frequencies of various sequence mutation classes. To exclude the effect of protein size [54], we normalized mutation frequencies by the background mutation rate or coding sequencing length. To distinguish between missense mutations with high (HiFI) or low (LoFI) functional impact, we used MutationAssessor [40], which is known to have high accuracy [55]. Loss of function (LOF) mutations were defined as the combination of nonsense and frameshift mutations. Benign mutations (as the combination of silent and LoFI) mutations reflect the background mutation rate of each gene. As a measure of the preferred occurrence of specific point mutations within a gene, termed 'mutation hot spots', we calculated entropy based 'mutation selection scores' as described [19].

#### Selection of features reliable for OG and TSG prediction

For the training of binary classifiers and for the selection of predictive features, we obtained OGs and TSGs from the Cancer Gene Census (CGC) [47] as well as NGs, as described [19]. To select features from the generated gene-alteration profiles that distinguish cancer drivers from NGs, we followed the methodology for parameter tuning from the TUSON (TUmor Suppressor and ONcogene) explorer [19] (Materials and Methods). We used the least absolute shrinkage and selection operator (Lasso) method [46] to identify the most reliable out of 48 parameters for predicting cancer genes.

**Figure 2:**
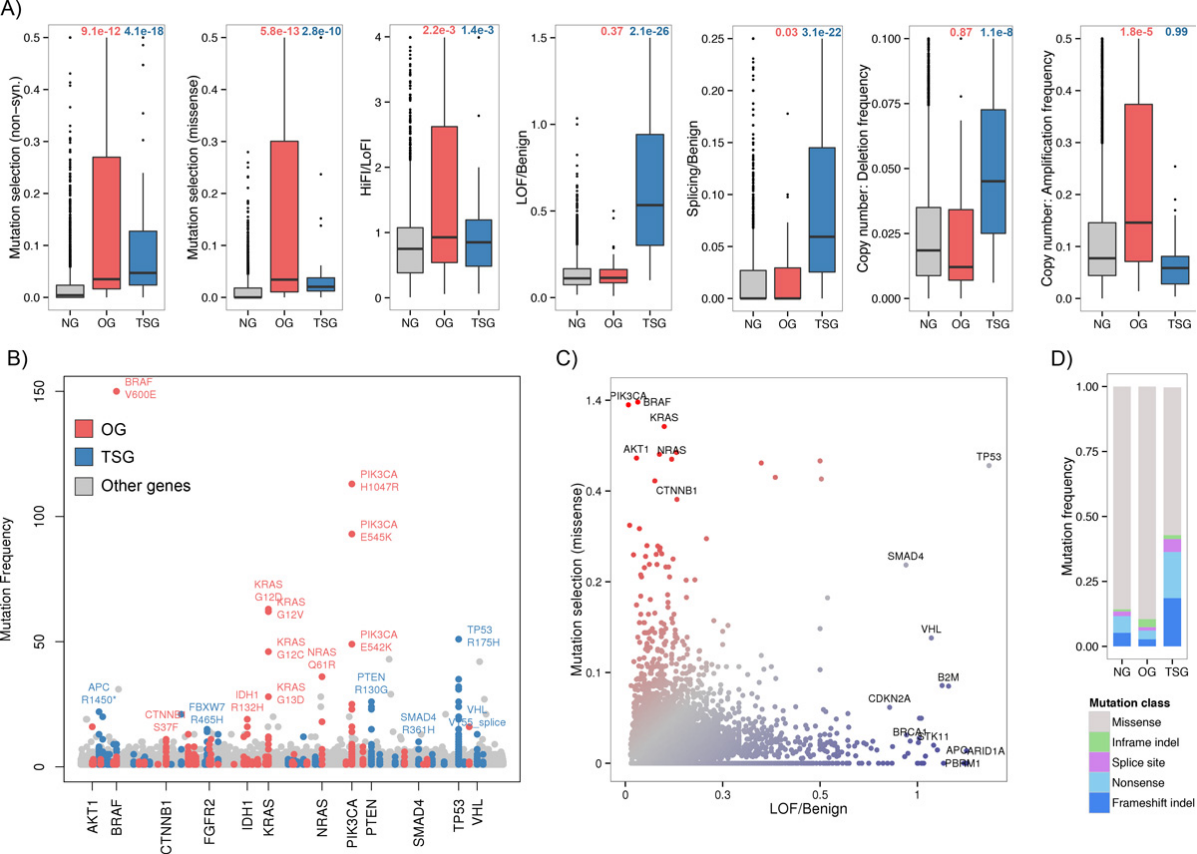
**Selecting features for OG and TSG prediction**. A) Box plots illustrate feature differences between OGs (red), TSGs (blue) and NGs (gray). Associated p-values on the top of each box plot are based on one-tailed Mann-Whitney U tests and reflect the differences between OGs and NGs, and TSGs and NGs. B) Dots reflect the frequencies of protein altering mutations in the combined set of tumors from seven cancer types. OGs (red), TSGs (blue) and NGs (gray) are sorted alphabetically on the x-axis. C) Proportions of loss of function (LOF) to benign mutations are plotted against the entropy based mutation selection scores for all human genes. Blue indicates high fractions of LOF mutations, while red indicates high mutation selection. D) Stacked bar plots present the relative frequencies of mutation classes in the combined tumor panel for OGs, TSGs and NGs.

For OG prediction, the most reliable parameters were the occurrence of mutation hot spots, represented by the mutation selection score (p = 5.8 × 10^−13^, one-tailed Mann-Whitney U test, β coefficient = 5.3), the ratio of HiFI to LoFI missense mutations (p = 2.2 × 10^−3^, β = 0.06), and the amplification frequency (p = 1.8 × 10^−5^, β = 2.48) (Figure 2A). These features indicate that canonical OGs are characterized by copy number amplifications or recurrent missense mutations with high impact on protein function. Examples for such somatic mutation hot spots include V600E in BRAF (265 tumors), H1047R in PIK3CA (113 tumors), or G12D in KRAS (63 tumors) (Figure 2B, Additional file 7). Overall, *BRAF *(S_m _= 2.71), *PIK3CA *(S_m _= 1.25), *KRAS *(S_m _= 1.11), and *IDH1 *(S_m _= 0.82) showed the highest selection scores for missense mutations (Sm) among OGs. Interestingly, known copy number driven OGs including *MYC *(S_m _= 0), *ALK *(S_m _= 0), and *SOX2 *(S_m _= 0) showed significantly low preference for mutation hot spots (p = 2.7 × 10^−18^, one-tailed Mann-Whitney U test comparing amplified versus non-amplified OGs based on CGC annotation). Notably, none of the expression parameters was selected as predictive feature (p > 0.4) implying that the OGs from the training set are not consistently over-expressed in cancer.

The most reliable feature set for TSG prediction included the ratio of LOF to benign mutations (p = 2.1 × 10^−26^, β = 2.13), splicing to benign mutations (p = 3.1 × 10^−22^, β = 1.85) and the frequency of homozygous copy number losses (p = 1.1 × 10^−8^, β = 1.08) (Figures 2A and 2D). In addition, given the significant underrepresentation of TSGs in amplicons, the Lasso approach also selected the amplification frequency as predictive (p = 2.8 × 10^−3^, β = −1.06). This indicates that canonical TSGs are characterized by copy number loss or mutations that have deleterious effects on protein function. Interestingly, multiple TSGs showed significantly recurrent LOF or splice site mutations including APC (R1450*; 22 tumors) and VHL (V155splice; 13 tumors) as well as missense mutations including TP53 (R175H; 51 tumors) and PTEN (R130G; 26 tumors) (Figure 2B, Additional file 7). Consequently, the selection scores for non-synonymous mutations (p = 4.1 × 10^−18^, β = 1.89) were high in the training set (Figures 2A and 2C) and thus selected for TSG prediction by Lasso. Expression parameters were not selected as reliable features (p > 0.4) for TSG prediction.

#### Applying cancer gene classifiers to ERGs reveals more TSGs than OGs

To uncover cancer drivers among ERGs, we applied the trained classification models that were used for feature selection. We used all 49 known OGs and TSGs for the training and feature selection step, because the size of the positive set was relatively small for machine learning. Therefore, the assessment of our predictors relied on 20-fold cross-validations instead of an independent test set. The average areas under the curve (AUC) as measure of prediction accuracy for OG and TSG classifications were 84.21% and 92.17%, respectively (Materials and Methods).

**Figure 3:**
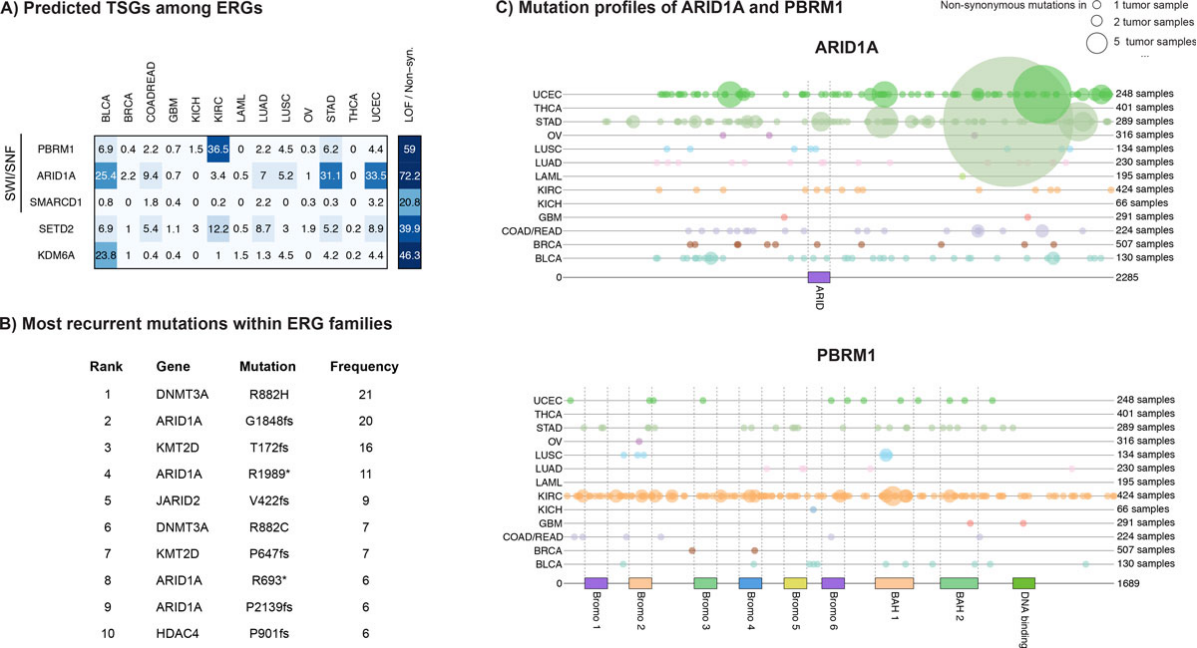
**Predicted TSGs and most recurrent mutations in the ERG family**. A) Predicted TSGs are listed along with the proportions of mutated samples in each indication and overall frequencies of LOF to benign mutations. B) Most recurrent mutations within the ERG family (del: deletion, *: nonsense mutation, fs: frameshift). C) Mutation profiles of ARID1A and PBRM1. Non-synonymous mutations are represented as solid circles, with color distinguishing different cancer types. Circle sizes are proportional to the mutation frequencies.

To identify cancer genes that are driven by mutation, we applied the predictors to the defined ERGs using the most predictive mutation parameters only. Overall five ERGs including the SWI/SNF complex subunits PBRM1, ARID1A, and SMARCD1 were classified as TSGs (Figure 3A). Exclusion of copy number data from the feature set yielded the same set. *ARID1A *had the highest ratio of LOF to benign mutations among ERGs, and was mutated in 25.4% of urothelial bladder tumors, 31.1% of gastric tumors, and 33.5% of endometrial tumors. Overall 72.2% of all non-synonymous mutations in ARID1A were LOF. *PBRM1 *was mutated in 36.5% of clear cell renal carcinomas, of which 75.0% were LOF. *SETD2 *was also classified as TSG with 39.9% of all non-synonymous mutations classified as LOF. Consistent with the mutation profiles of TSGs in the training set, multiple LOF mutations had a non-random distribution within predicted TSGs in more than one tumor (Figures 3B and 3C). ARID1A, for example, showed a frameshift mutation at position 1848 in 20 tumors (Figure 3C). Overall, with the exception of alterations in *DNMT3A*, the most recurrent mutations within ERG families were associated with loss of function (Figure 3B).

Using the missense mutation selection score and the ratio of HiFI to LoFI mutations, only *DNMT3A *was predicted as OG. This result reflects the lack of recurrent and potentially activating hotspot missense mutations within ERG families in our tumor panel. With the exception of DNMT3A, we detected nonsense mutations and indels, but no missense point mutations among ERGs that occurred in more than four tumors. The driver classification of DNMT3A by our OG predictor can be attributed to the occurrence of a mutation hot spot in acute myeloid leukemia. In total 28 (14.4%) of the 195 tumors showed a missense mutation on position 882 resulting in an overall mutation selection score of 0.55.

We expected EZH2 to be classified also as an OG, since it is a validated target pursued by multiple pharmaceutical companies. Activating mutations within the SET domain of EZH2 are frequent in non-Hodgkin's lymphoma [56], but were not found as recurrent in the analyzed cancer types.

With copy number data as an additional feature, completing the set of selected predictive parameters, *ACTL6A and ATAD2 *were the only predicted OG among ERGs. However, since the amplified genomic regions harboring these genes were typically very large, with an average length exceeding 50 Mb, it is equally likely that both genes are only amplified as a passenger genes.

### Detection of ERGs with consistent over- or under-expression in cancer

The Lasso-based feature selection for OG/TSG prediction showed that canonical cancer drivers are usually characterized by significant mutation patterns or copy number alterations (Figure 2A), but not by consistent gene expression patterns. Consequently, while our machine learning approach enabled us to uncover cancer driver-like mutation and copy number alterations among ERGs, significant gene expression patterns could not be detected by prediction.

**Figure 4:**
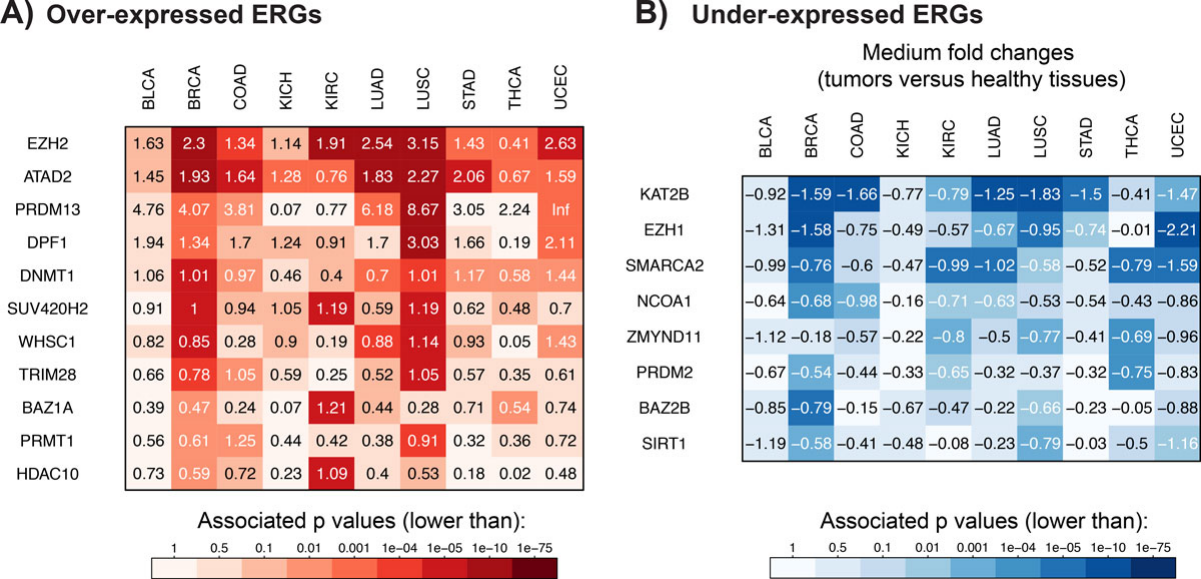
**Epigenetic regulators with significant gene expression profiles in cancer**. Significantly (A) over- and (B) under-expressed ERGs are ranked according to the combined p-values (based on Fisher's probability test) over all cancer types. Numbers reflect log2-fold changes, while colors reflect associated p-values. ERGs with consistently over-expression in tumors included ***EZH2 ***(p**_F _**= 3.2 × 10**^−112^**), ***ATAD2 ***(p**_F _**= 1.9 × 10**^−76^**), ***PRDM13 ***(p**_F _**= 2.7 × 10**^−27^**), ***DPF1 ***(p**_F _**= 1.0 × 10**^−19^**), ***DNMT1 ***(p**_F _**= 8.3 × 10**^−19^**), ***SUV420H2 ***(p**_F _**= 1.7 × 10**^−15^**), ***WHSC1 ***(p**_F _**= 3.3 × 10**^−15^**), ***TRIM28 ***(p**_F _**= 1.2 × 10**^−8^**), ***BAZ1A ***(p**_F _**= 2.2 × 10**^−7^**), ***PRMT1 ***(p**_F _**= 9.6 × 10**^−6^**), and ***HDAC10 ***(p**_F _**= 8.1 × 10**^−5^**). ERGs with consistently lower expression in tumors included ***KAT2B ***(p**_m _**= 1.0 × 10**^−74^**), ***EZH1 ***(p**_m _**= 2.3 × 10**^−42^**), ***SMARCA2 ***(p**_m _**= 2.0 × 10**^−25^**), ***NCOA1 ***(p**_m _**= 1.2 × 10**^−10^**), ***ZMYND11 ***(p**_m _**= 3.6 × 10**^−9^**), ***PRDM2 ***(p**_m _**= 9.5 × 10**^−7^**), ***BAZ2B ***(p**_m _**= 3.5 × 10**^−6^**) and ***SIRT1 ***(p**_m _**= 8.1 × 10**^−6^**).

**Figure 5:**
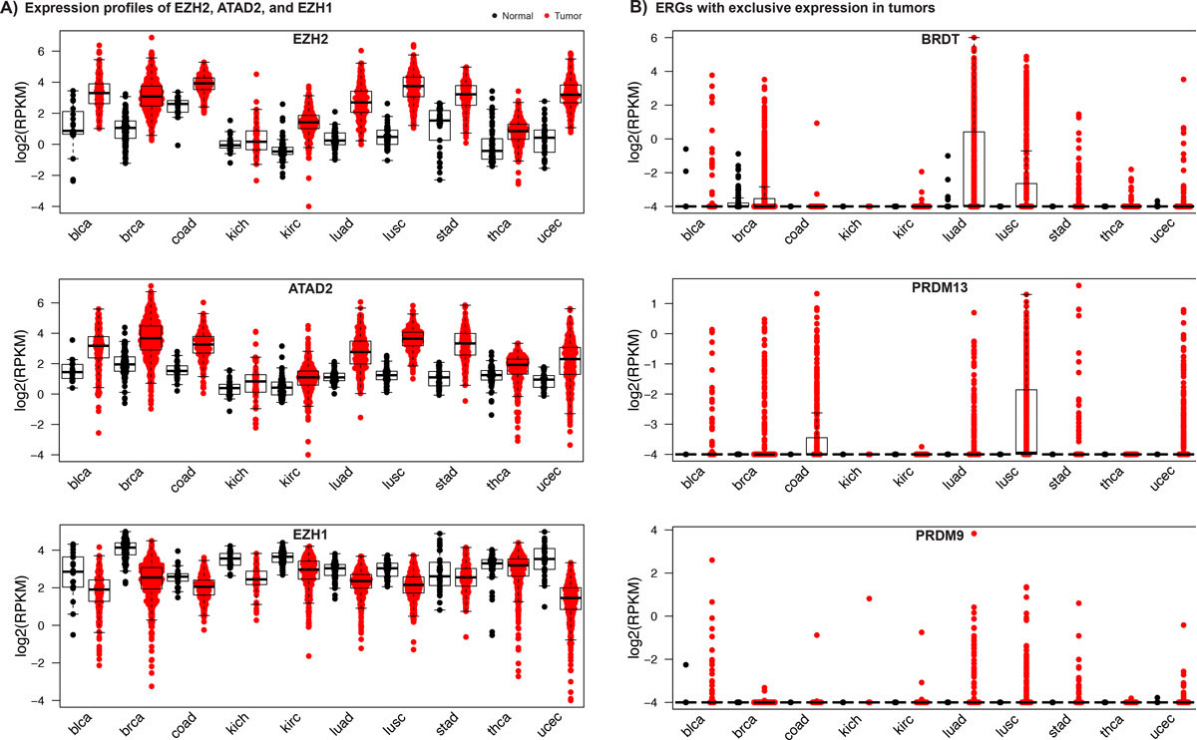
**Expression plots of significantly expressed ERGs**. Gene expressions of (A) ***EZH2***, ***ATAD2***, ***EZH1***, and (B) ***BRDT***, ***PRDM13 ***and ***PRDM9 ***are shown in RPKM units (black: healthy tissues, red: tumors). Gene expression levels are reflected by RPKM values.

To pinpoint ERGs with consistently higher or lower expression in cancer, we determined the differential expression significance across the ten cancer types with available RNAseq data using negative binomial generalized linear models (GLM) [45] (Materials and Methods). To assess the overall significance of differential expression in cancer for each gene, we combined the p-values resulting from the cancer type specific analyses using Fisher's probability test. Overall 11 ERGs showed consistent up-regulation in all cancer types with combined p-values (p_F_) lower than 0.001 (Figure 4A, Additional file 8). *EZH2 *showed the most significant over-expression in tumors (p_F _= 3.18 × 10^−112^) (Figure 5A) not correlating with copy number alterations (p = 0.87 based on linear regression between RPKM expression levels and total copy number) (Materials and Methods). The *MYC *cofactor [57,58] bromodomain reader *ATAD2 *(ATPase family, AAA domain containing 2) was the second most significantly over-expressed ERG (p_F _= 2.6 × 10^−25^). Expression levels of *ATAD2 *correlated significantly with copy number changes (p = 3.1 × 10^−12^) consistent with the length of the *MYC *amplicon that spans the genomic region of *ATAD2 *in 98% of the cases. Other ERGs with homogeneous over-expression in cancer included *PRDM13*, *DPF1*, *DNMT1*, *SUV420H2*, *WHSC1*, *TRIM28*, *BAZ1A*, *PRMT1*, and *HDAC10*.

Quantile-based differential expression analysis (Materials and Methods) revealed that *BRDT*, *PRDM9*, and *PRDM13 *were exclusively expressed in tumors but not in paired healthy tissues (Figure 5B). With high expression levels across several cancer types, the testis-specific *BRDT *gene [59,60] displayed characteristics of a cancer-testis (CT) gene. CT genes are genes with normal expression restricted to adult testicular germ cells, and yet are aberrantly activated and expressed in various cancer types [61]. As such CT genes are interesting targets in cancer therapy. As previously observed in non-small cell lung cancers [62], differential expression analysis between *BRDT *expressing and non-expressing LUSC tumors revealed co-expression with canonical CT genes such as *MAGE-A11*, *GAGE4*, *GAGE5*, *GAGE6 *and *GAGE12I *(Additional file 9, Materials and Methods). Almost all co-expressed genes also showed tumor-specific expression in LUSC. *PRDM9 *and *PRDM13 *also showed exclusive expression in cancer, but their biological roles in these tumors are unclear.

Consistently down-regulated ERGs included *KAT2B*, *EZH1*, *SMARCA2*, *NCOA1*, *ZMYND11*, *PRDM2*, *BAZ2B *and *SIRT1*, which showed significantly lower expression (p_F _< 0.001) in tumors compared to healthy tissues (Figure 4B). Comparing the sets of over- or under-expressed ERGs showed that closely related genes such as *KAT2A *and *KAT2B *exhibited different expression profiles. EZH2 and EZH1, for example, form PRC2 (Polycomb repressive complex 2) complexes with similar functionalities [63], but opposite expression profiles. As another example, while *PRDM9 *and *PRDM13 *were exclusively expressed in tumors, *PRDM2 *was consistently down-regulated in tumors.

Taken together the resulting panel of significantly over- or under-expressed ERGs form an interesting candidate set of genes that potentially drive the development of cancer via dysregulated expression. This model is generally not applicable to classical OGs and TSGs, but might hold true for ERGs.

### Co-expression network analysis

In addition to the identification of significant expression patterns in tumors, we used the expression levels in healthy tissues to detect co-expressed genes under normal conditions. The main objective of this analysis was to uncover the involvement of ERGs in co-expression networks, which frequently form jointly regulated functional modules [64]. Co-expressed genes can have similar biological activities and even physically interact. In some cases co-expression may reflect that one gene encoding protein regulates the activity of the other gene.

Using variance stabilized TCGA RNAseq count data as a measure of gene expression, we analyzed co-expression networks by estimating pairwise linear relationships between all protein coding human genes (Materials and Methods).

**Figure 6:**
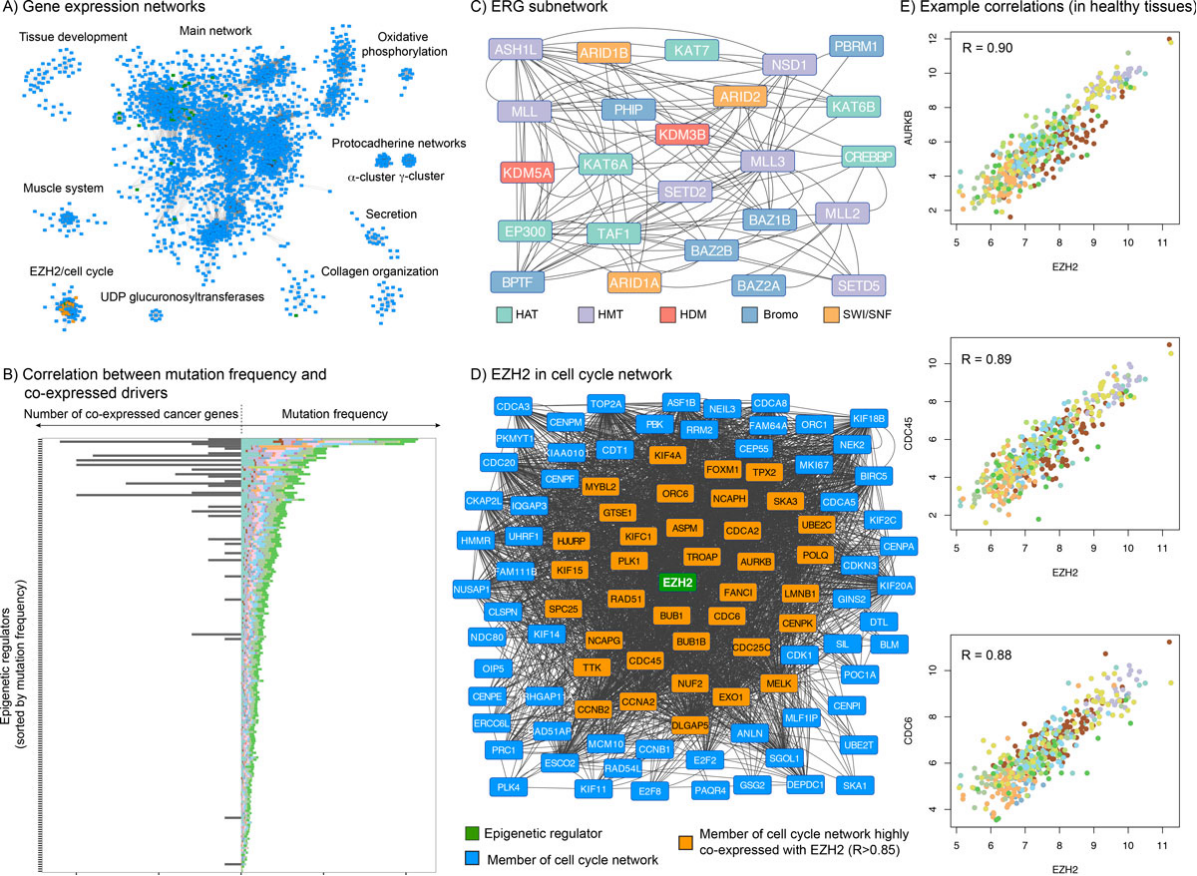
**Co-expression network analyses**. A) Using Cytoscape co-expressed genes are visualized as networks with nodes representing genes and edges reflecting pairwise co-expression relationships in healthy tissues. B) Numbers of co-regulated cancer genes in healthy tissues (right panel of the plot) are shown along with the mutation frequencies (left panel of the plot). Mutation frequencies are presented as stacked bars with cancer type dependent coloration. ERGs are sorted on the y-axis by the overall mutation frequencies. C) A subnetwork within the main co-expression network contains 24 co-expressed ERGs. Colors indicate the corresponding ERG families. D) EZH2 (green) and 99 co-expressed genes form one co-regulated network that is significantly enriched for cell cycle regulators. Genes that are directly connected with EZH2, because they show a very high degree of co-expression (R > 0.85), are highlighted in orange. Genes that are present in the network, but not directly connected with EZH2 are shown in blue. F) Examples of positive correlations between EZH2 and co-expressed cell cycle regulators. Each dot reflects the gene expression level (represented by variance stabilized RNAseq count data) of EZH2 (x axis) and the co-expressed gene (y axis). Dots are colored according to tissue type.

Based on hard thresholding (r > 0.85) we converted the resulting 19,115 × 19,115 similarity matrix into an adjacency matrix, which contains binary information (0: no co-expression; 1: co-expression) about pairwise co-expression. Transforming the adjacency matrix into nodes (genes) and edges (co-expression) resulted in one major network with 2465 genes including 37 ERGs, and 11 separate networks with 8 to 112 genes (Figure 6A).

#### EZH2 is a member of a cell cycle network

Using gene ontology enrichment (Materials and Methods) we found that the discrete co-expression networks, which were not connected to any node of the main network, were associated with specific biological functions such as muscle contraction (p_GO _= 8.9 × 10^−11^), collagen fibril organization (p_GO _= 4.4 × 10^−10^), tissue development (p_GO _= 1.4 × 10^−5^), oxidative phosphorylation (p_GO _= 3.1 × 10^−24^), or regulation of secretion (p_GO _= 2.3 × 10^−3^) (Additional file 10). Two co-expression modules contained 37 members of the protocadherin family representing tightly linked gene clusters α and γ, consequently associated with cell-cell adhesion (p_GO _= 2.7 × 10^−34^, 1.8 × 10^−25^). Another network included nine members of the UDP glucuronosyltransferase 1 family, significantly associated with various metabolic processes.

Strikingly, we identified a distinct network with 100 genes, almost exclusively associated with the regulation of the cell cycle (p_GO _= 4.4 × 10^−57^) (Figure 6D). *EZH2 *is the only epigenetic regulator in this module. In total 62 genes in the network were annotated cell cycle regulators with consistent up-regulation in tumors, including cell division cycle genes *CDC6*, *CDC45*, and *CDC25C*, cyclins *CCNA2, CCNB1 *and *CCNB2*, genes encoding for aurora kinase B (AURKB) and its interaction partner NUF2, mitotic checkpoint protein kinase TTK, RAD51, checkpoint activator FANCI, DLGAP5, polo-like kinases (PLK) 1 and 4 along with interacting cyclin regulator FOXM1. Other essential cell cycle genes included *BUB1*, *BUB1B*, *CHEK2, CDK1*, and several members of the kinesin family.

Multiple members of the cell cycle network are known to regulate or physically interact with each other. For example, the expression of EZH2 is known to be regulated by the co-expressed transcription factor *E2F2 *[65]. The exact role of EZH2 as the only ERG in the cell cycle network, however, is not clear.

#### Co-expression patterns in the main network

Analyzing the main network revealed multiple pairwise co-expressions between ERGs and cancer genes. While we took only a subset of genes of the CGC to train our predictors, we defined all genes from the CGC as cancer drivers in the co-expression analysis. Some examples for positive correlations between ERGs and drivers are illustrated in Figure 6E and Additional file 11. Interestingly, genes encoding longer proteins showed more co-expressed genes, presumably because they provide increased surfaces for interaction. Consequently, without normalizing for coding sequence length, frequently mutated ERGs correlated with more cancer genes than rarely mutated ERGs (p = 2.3 × 10^−5 ^using permutation test) (Figure 6B).

Overall, we found seven cases, where the expression of one ERG was negatively correlated with the expression of another gene (R < −0.85) (Additional file 12). The transcription factor *BUD31 *was involved in three of the seven instances including *ASH1L*, *KAT6A*, and *KDM3B*. Without known functional causalities, however, it is difficult to interpret these negative correlations.

Identifying sub-networks by investigating highly co-expressed gene pairs (directly linked nodes) within the major network revealed 24 inter-connected co-expressed ERGs (Figure 6C). This sub-network was composed of members from different ERG families. Similar to all observed co-expression patterns, this finding may not only imply common functionality, but also reflect that the epigenetic machinery is partially controlling itself or is commonly controlled by another regulatory mechanism.

### Proteomic analysis of the antiproliferative effect of EZH2 inhibition in mutant lymphoma cells

While the exact role of *EZH2 *in the identified cell cycle network is not clear, *EZH2 *is known as direct transcription repressor or activator of several cell cycle regulators (Additional file 13). As member of the Polycomb-group family, EZH2 acts as transcription repressor of several cell cycle-related tumor suppressor genes such as CDKN1C through methylation of histone H3 on lysine 27 (H3K27) [66,67]. In an alternative model for EZH2 mediated regulation, EZH2 promotes tumorigenicity by direct activation of OGs such as STAT3 [68].

Inhibition of EZH2 has been suggested to induce cell cycle arrest in G1 phase and antiproliferative response in the mutant-bearing lymphoma cell line WSU-DLCL2 (EZH2^Y641F^) [13]. The associated study further showed that proliferation of EZH2 wildtype cells was not affected by the same treatment. After only 2 days of compound treatment cell cycle genes were found significantly down-regulated in the mutant cell line based on microarray experiments. Overall, we identified 11 out of the 30 most down-regulated cell cycle genes from this study in our co-expressed network (*CDC6*, *BUB1*, *CDC25C*, *BUB1B*, *TTK*, *CCNB1*, *CCNA2*, *PKMYT1*, *E2F2*, *CDC20*, *PLK1*).

To analyze the effect of EZH2 inhibition on the proteome, we treated WSU-DLCL2 cells with the selective EZH2 small molecule inhibitor EPZ-6438 (Epizyme^®^, Cambridge, MA) [69,70] and measured global proteomic changes after 2, 4, 6 and 8 days using SILAC (stable isotope labeling by amino acids in cell culture) based mass spectrometry (Materials and methods).

Consistent with previous findings [13], we observed decreased viability of WSU-DLCL2 cells after EZH2 inhibition. Concordant with EZH2 as member of the PRC2 complex, which trimethylates histone 3 on lysine 27, the level of the H3K27me3 histone mark decreased by a factor of 2 and 3.3 after 2 and 5 days respectively (Additional file 14).

We identified 2530 proteins on average and quantified their intensity changes between EPZ-6438 treated cells (heavy labeled) and their respective non-treated control cells (light labeled) (Additional file 15). The combined proteome profiles over all time points comprised 3066 proteins. Overall 1852 proteins were commonly identified in all time point experiments. Clustering the associated time courses revealed three different profiles representing up-, down-, and non-regulated proteins (Additional file 16).

**Figure 7:**
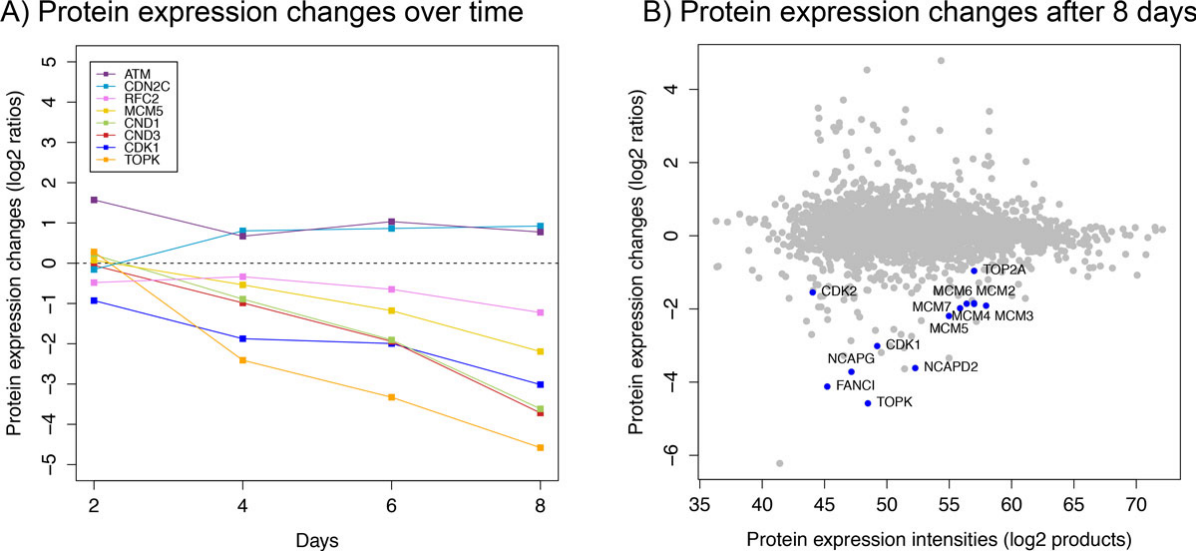
**Quantitative mass spectrometry based proteomic analysis after EZH2 inhibition**. A) Protein expression differences of selected cell cycle regulators in EPZ-6438 (Epizyme^®^, Cambridge, MA) versus DMSO treated lymphoma cells (WSU-DLCL2) are represented as log2 ratios. B) Global protein expression changes after 8 days of EPZ-6438 treatment. Down-regulated cell cycle regulators are highlighted in dark blue.

In total 267 and 202 proteins showed minimum 2-fold increase or decrease in expression levels respectively. Based on gene ontology (GO) analysis, the set of down-regulated proteins was significantly enriched for genes associated with cell cycle (p = 2.25 × 10^−9^) and DNA replication (p = 6.97 × 10^−17^) (Additional file 17). Among the 59 down-regulated cell cycle proteins were CDK2, CND1, MCM7, RFC2 and several regulators that were co-expressed in the EZH2 cell cycle network including CDK1, CND3, FANCI, BUB1, KIF11, TOP2A, TOPK, and UHRF1 (Figures 7A,B). Overall, 24 cell cycle associated proteins were up-regulated after EZH2 inhibition including tumor suppressors ATM, BRCA2 and cell cycle inhibitor CDN2C.

Our proteomics results do not distinguish whether EZH2 inhibition acts directly on the expression of cell cycle genes or more generally induces cell cycle arrest. However, coupled with the known regulatory roles of EZH2 as a member of the PRC2 complex and results from previous studies [13], these data suggest a regulatory function of EZH2 in controlling it's co-expressed cell cycle network.

## Discussion

Alterations in the epigenetic machinery that lead to uncontrolled cellular proliferation have become an important research topic in the field of oncology. By training cancer gene predictors based on TCGA data, we found that classical cancer drivers are characterized by significant mutation or copy number patterns, but not by altered expression. Among ERGs we identified multiple TSGs with significant proportions of loss of function mutations. Given the lack of recurrent mutation hot spots within the ERG families in the tumor panel, DNMT3A was the only ERG that showed an OG-like alteration profile. However, whether DNMT3A acts as OG or TSG has been debated, and additional studies are required to understand the exact role of DNMT3A in cancer. The classification of *DNMT3A *as OG driver can be attributed to the identification of a mutation hot spot on position 882 in acute myeloid leukemia. This shows that our predictor, which was trained on the combined set of all tumors, was capable to detect significant alterations within a single cancer type. It also makes clear, however, that the approach is biased towards included cancer types. Activating mutations within the catalytic SET domain of EZH2, for example, are known in non-Hodgkin's lymphoma [71], but were absent in our tumor cohort.

Many ERGs, which were not predicted as drivers, had dysregulated expression in cancer. The role of dysregulated genes in cancer is generally difficult to determine [72], but the discovered expression profiles among ERGs were remarkable. *EZH2 *was the most significantly up-regulated gene. Strikingly, co-expression network analysis uncovered EZH2 as the only ERG in a co-expressed cell cycle module. Selective inhibition of EZH2 has been shown to decrease expression of multiple cell cycle regulators [13], many of which are in our co-expressed network. Despite the limitations of mass spectrometry to identify a subset of the whole human proteome only, we confirmed the down-regulation of cell cycle proteins and showed a decrease of the PRC2-associated H3K27 methylation mark after EZH2 inhibition. We cannot determine, however, whether EZH2 inhibition acts directly on the expression of cell cycle genes or more generally induces cell cycle arrest. Interestingly, the EZH2 homolog EZH1 has been proposed to form PRC2 complexes with similar functions [63], EZH1 is commonly down-regulated in cancer, which contradicts its involvement as a cell cycle promoting PRC2 subunit.

Several other ERGs were significantly over-expressed in tumors. *BRDT*, *PRDM9 *and *PRDM13*, for example, were exclusively expressed in tumors. BRDT showed characteristics of a CT gene, and was co-expressed with other known CT genes. The underlying mechanisms that induce the co-expression of these genes or the effect on the cancer cell are not known, but BRDT may present a potential candidate for initializing their expression as an epigenetic regulator.

While overexpression of *ATAD2 *and *ACTL6A *are related to their genomic locations on large frequently amplified chromosome regions, *DNMT3B, KAT2A, SUV420H2 *and several other ERGs showed ubiquitous significant up-regulation in cancer, therefore presenting an interesting candidate set for potential therapeutic targets.

Taken together, our prediction method identified several ERGs with mutation alteration profiles characteristic of classical TSGs. *DNMT3A *was the only predicted OG-like ERG with mutation hot spots in acute myeloid leukemia. Expression analysis further supports the role of EZH2 as an OG. Our study provides the first systematic analysis of the epigenetic regulators, thus providing basis for further prioritization of such players as candidates for therapeutic target discovery.

## Competing interests

All authors were employed by Genentech, Inc. during the time the study was done.

## Authors' contributions

FG and ZZ were responsible for the study design. FG performed data analysis. SD and DA performed proteomic analysis. FG, GM and ZZ wrote the manuscript.

## Supplementary Material

Additional file 1 **Epigenetic regulators of gene expression as writers, erasers and readers of covalent DNA and histone modifications**. The upper panel provides an overview of writers (DNMTs, HATs, and HMTs), erasers (DNDMTs, HDACs, and HDMTs), and readers (bromo domain containing and methyl binding proteins) of epigenetic marks. Epigenetic regulators can be identified by the presence of specific associated domains, which are listed on the right of the lower panel. The sequence similarities between contained domains or total protein sequences formed the phylogenetic trees for each epigenetic gene family as shown on left. Click here for file

Additional file 2 **Description of genomic features**. Click here for file

Additional file 3 **List of members of ERG families**. Click here for file

Additional file 4 **Illustration of the SWI/SNF complex**. Click here for file

Additional file 5 **Overview: Cancer gene prediction applied to ERGs**. Click here for file

Additional file 6 **Genomic alterations of HATs, HDACs, HMTs, HDMs and members of the SWI/SNF complex**. The compositions of the plots are explained in Figure 1. Click here for file

Additional file 7 **List of frequent mutations in TCGA**. Click here for file

Additional file 8 **Differential gene expression analysis results**. Click here for file

Additional file 9 Co-expression of cancer testis genes. A) Volcano plot resulting from the differential expression analysis between BRDT expressing and BRDT non-expressing LUSC tumors. B) Gene expression levels of co-expressed cancer testis in LUSC (black: healthy tissues, red: tumors). Click here for file

Additional file 10 **Gene ontology enrichment analysis of identified co-expression networks**. For some of the identified networks, gene ontology enrichment analyses were performed. "X" is the total number of annotated genes in the given network, while "x" is the number of annotated genes in the network that are associated with the given gene ontology accession (GO-ID). "N" is the number of annotated genes in the background set, while "n" is the number of genes from the background set that are associated with the given gene ontology accession (GO-ID). Click here for file

Additional file 11 Examples of co-expression between ERGs and other genes in healthy tissues. Each dot reflects the gene expression levels (represented by variance stabilized RNAseq count data) of the ERG (x axis) and the co-expressed gene (y axis). Dots are colored according to the associated tissue indication. Click here for file

Additional file 12 Negative correlations between expression levels of ERGs and other genes. Analogously gene expression (variance stabilized RNAseq count data) of the epigenetic regulator (x axis) and the co-expressed gene (y axis). Colors indicate the associated tissue indication. Click here for file

Additional file 13 Known models for EZH2 as cell cycle regulator. Two established models describe a cell cycle regulating role of EZH2: With its transcription repressing role as member of PRC2 complex (left panel), EZH2 enhances the expression of cell cycle regulators indirectly by repressing associated tumor suppressors such as CDKN1C. In an alternative model, EZH2 acts as a direct activator (right panel). Phosphorylated EZH2 activates STAT3 via methylation, which in turn activates the cyclin D1/CDK2 complex. Interestingly CDK1 and CDK2 have been shown to phosphorylate EZH2. In addition EZH2 has been shown to inhibit BRCA1 phosphorylation presumably via interaction with Akt-1 resulting into increase of cell cycle promoting CDC25C. Click here for file

Additional file 14 **Mass spectrometry based quantitation of H3K27me following EZH2 inhibition**. Click here for file

Additional file 15 Mass spectrometry results. List of identified proteins and corresponding quantitative results. Click here for file

Additional file 16 Clustered time series. Using fuzzy c-means clustering, time course profiles formed three clusters of down-, up-, and non-regulated proteins. Colors reflect the similarities between specific time series and the associated cluster. Click here for file

Additional file 17 **Gene ontology enrichment analysis of regulated proteins**. Click here for file
