# Supplementary figures and images for: Bioinformatics analysis of thousands of TCGA tumors to determine the involvement of epigenetic regulators in human cancer
Source: BMC Genomics. 2015 Jun 18;16(Suppl 8):S5. doi: 10.1186/1471-2164-16-S8-S5 (PMC4480953; doi:10.1186/1471-2164-16-S8-S5)

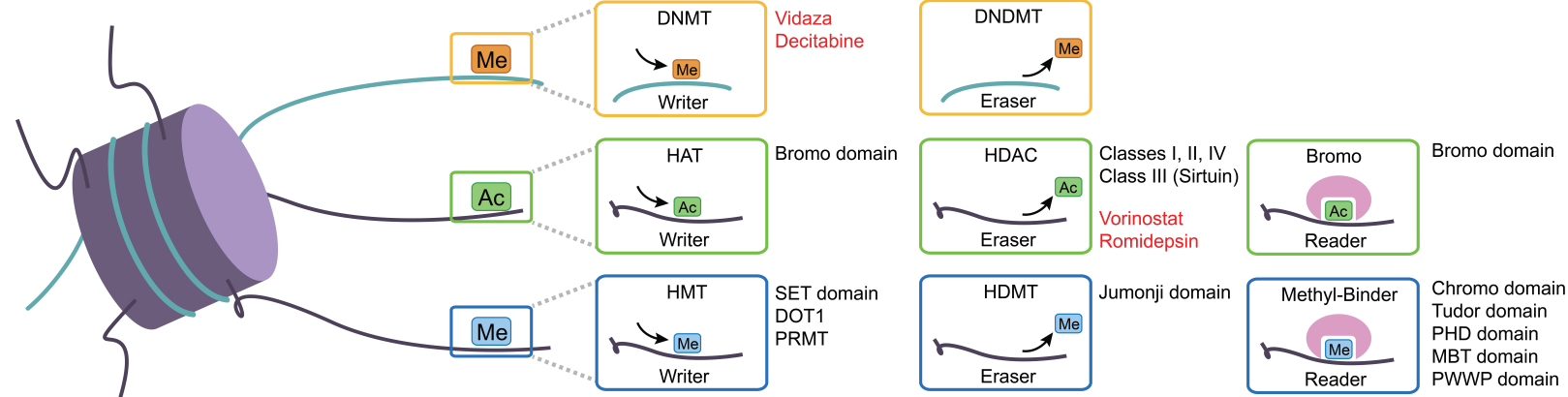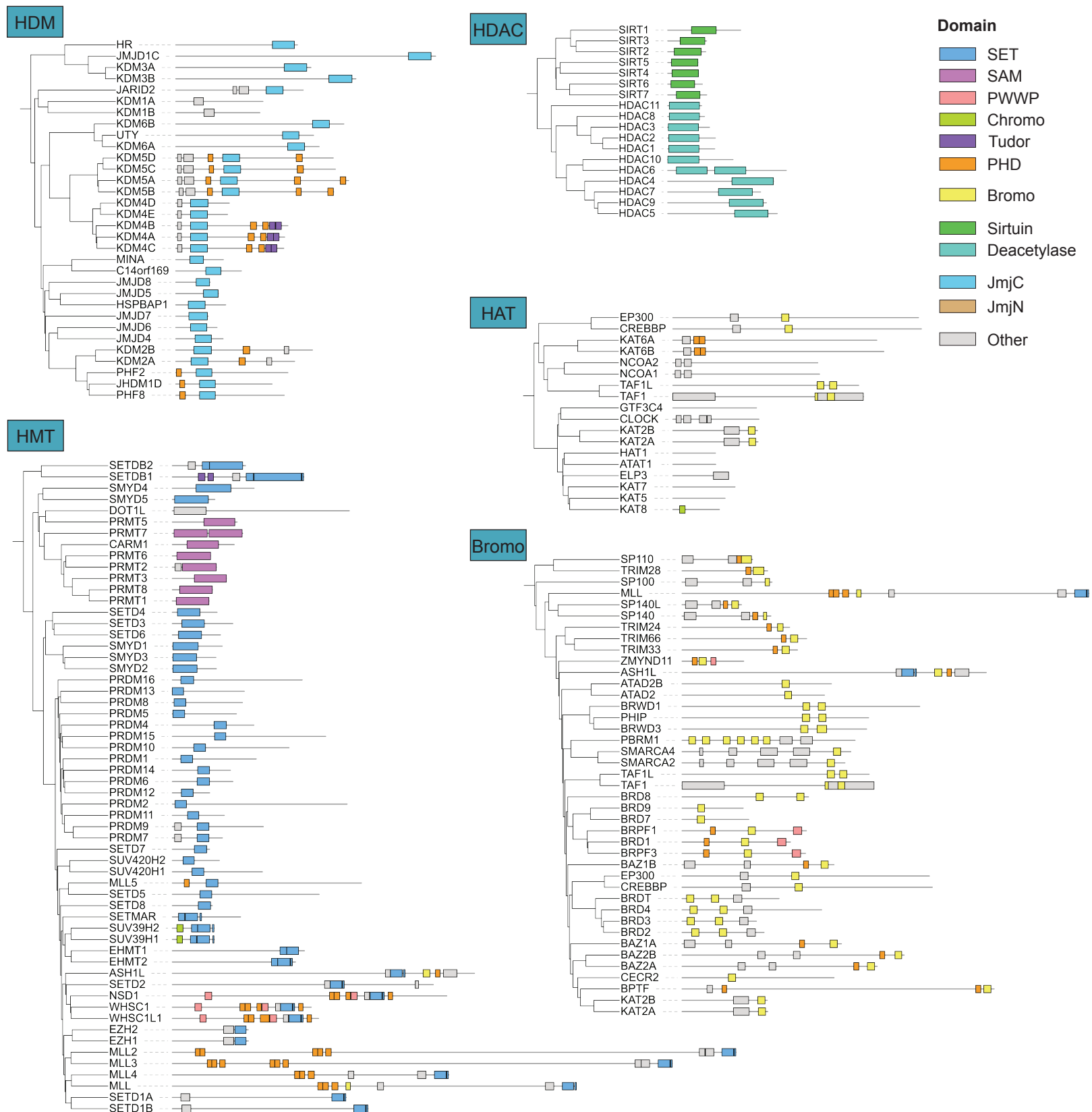

Supplement: Additional file 1 — Epigenetic regulators of gene expression as writers, erasers and readers of covalent DNA and histone modifications. The upper panel provides an overview of writers (DNMTs, HATs, and HMTs), erasers (DNDMTs, HDACs, and HDMTs), and readers (bromo domain containing and methyl binding proteins) of epigenetic marks. Epigenetic regulators can be identified by the presence of specific associated domains, which are listed on the right of the lower panel. The sequence similarities between contained domains or total protein sequences formed the phylogenetic trees for each epigenetic gene family as shown on left. [file 1471-2164-16-S8-S5-S1.pdf]

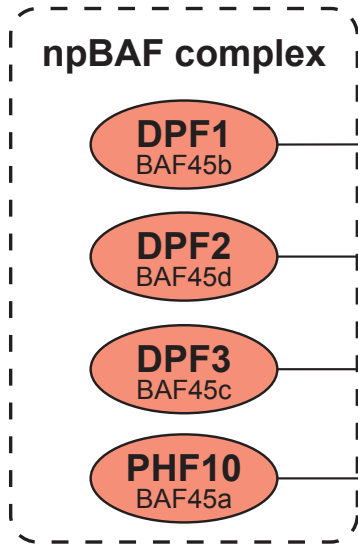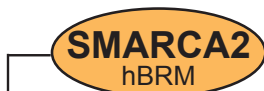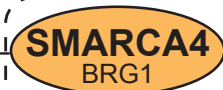

**PBAF complex**

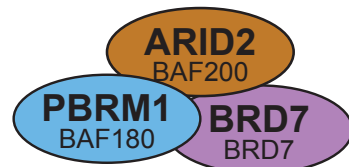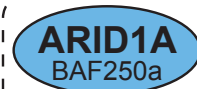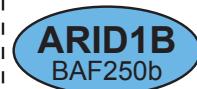

**BAF complex**

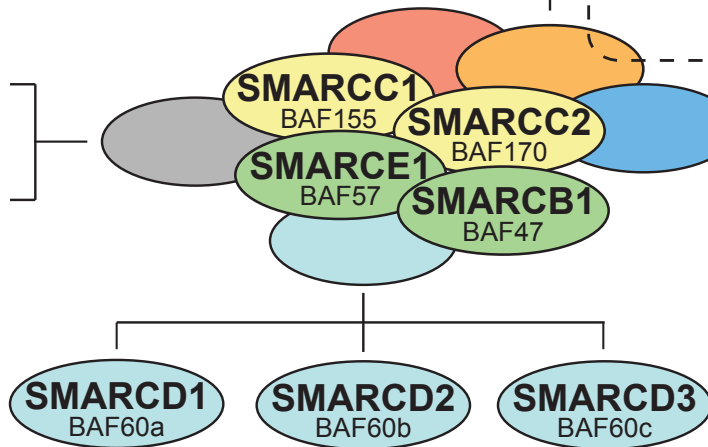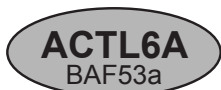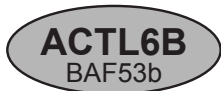

Supplement: Additional file 4 — Illustration of the SWI/SNF complex. [file 1471-2164-16-S8-S5-S4.pdf]

# Deacetylases

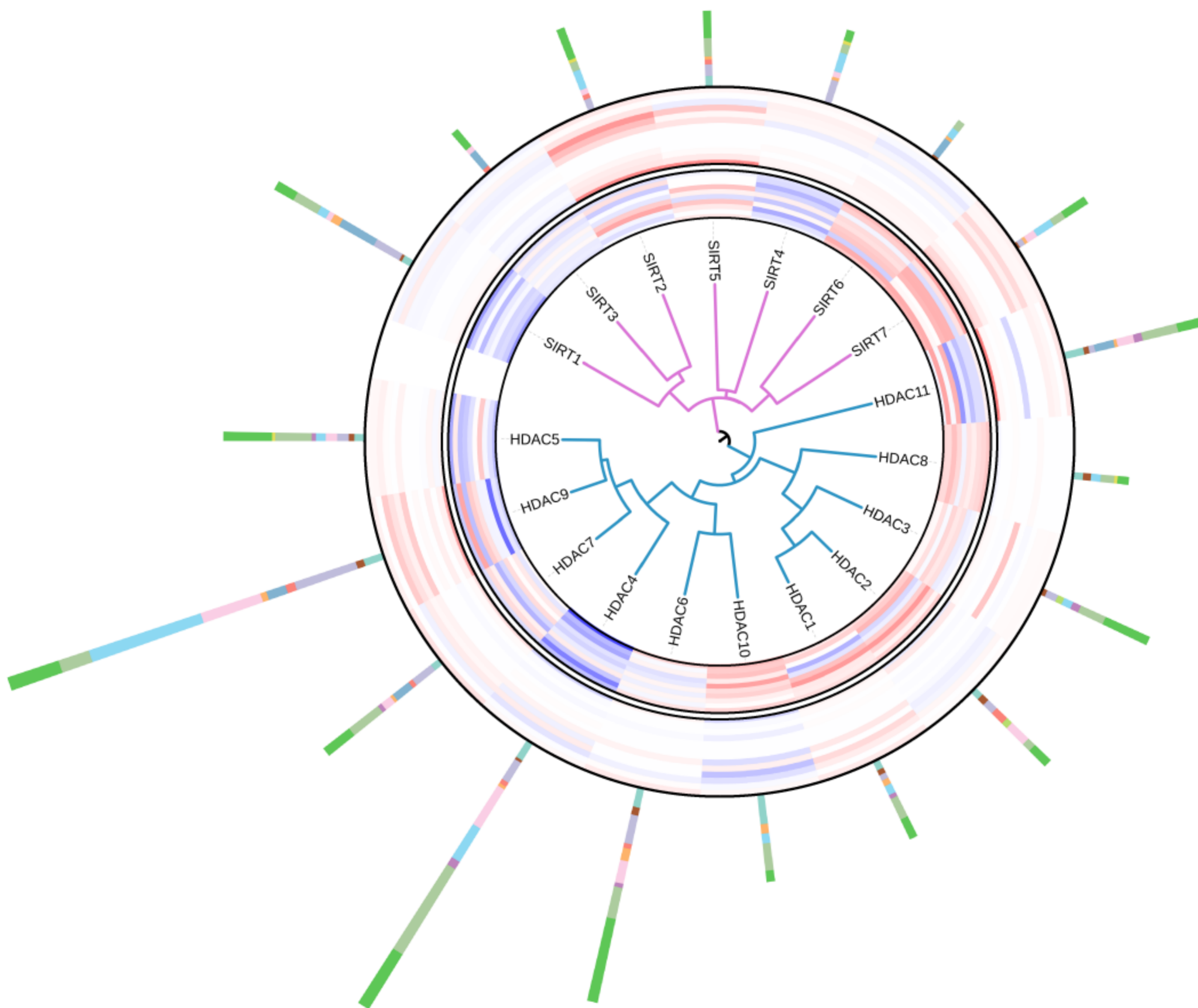

# Acetyltransferases

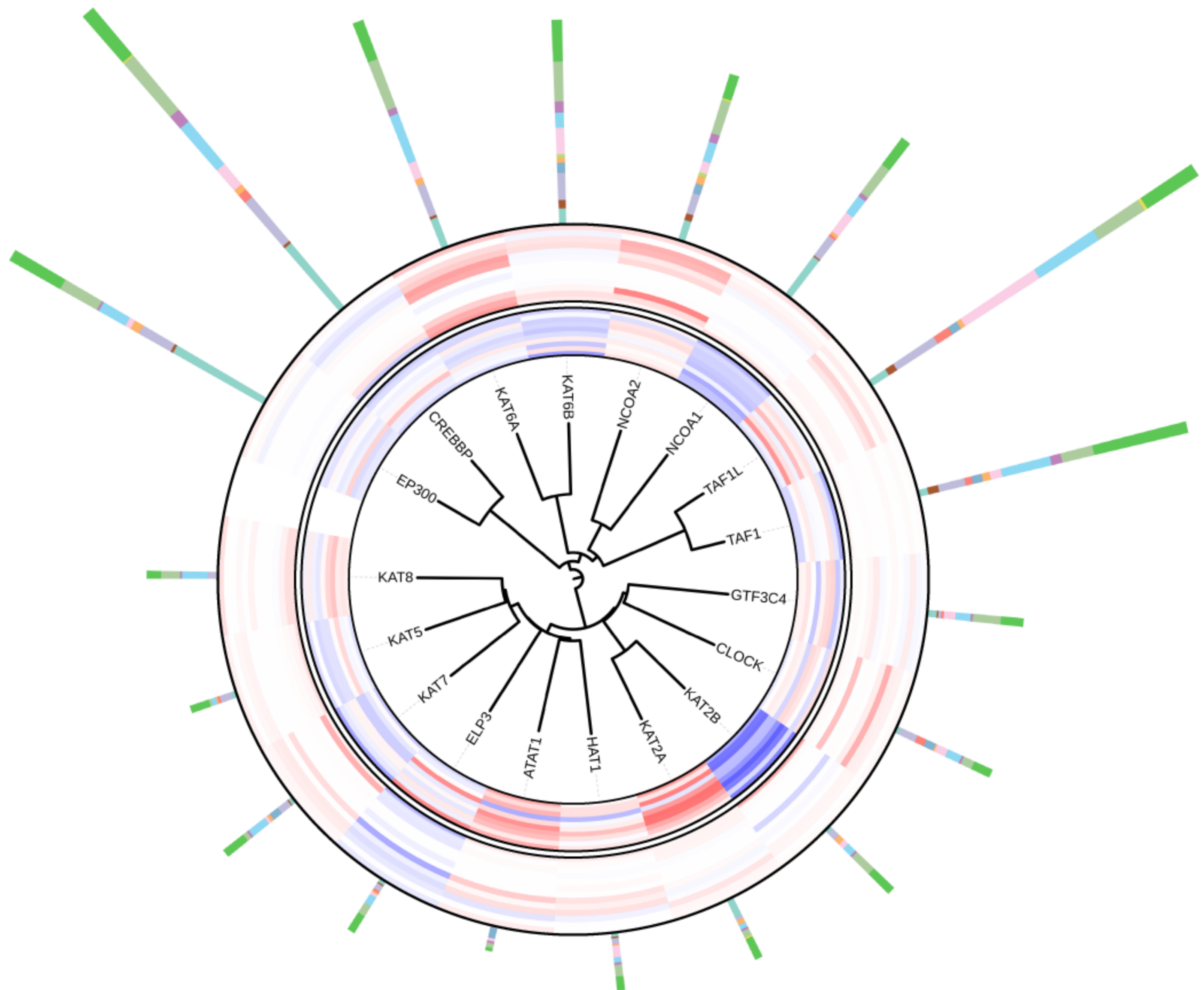

# Demethylases

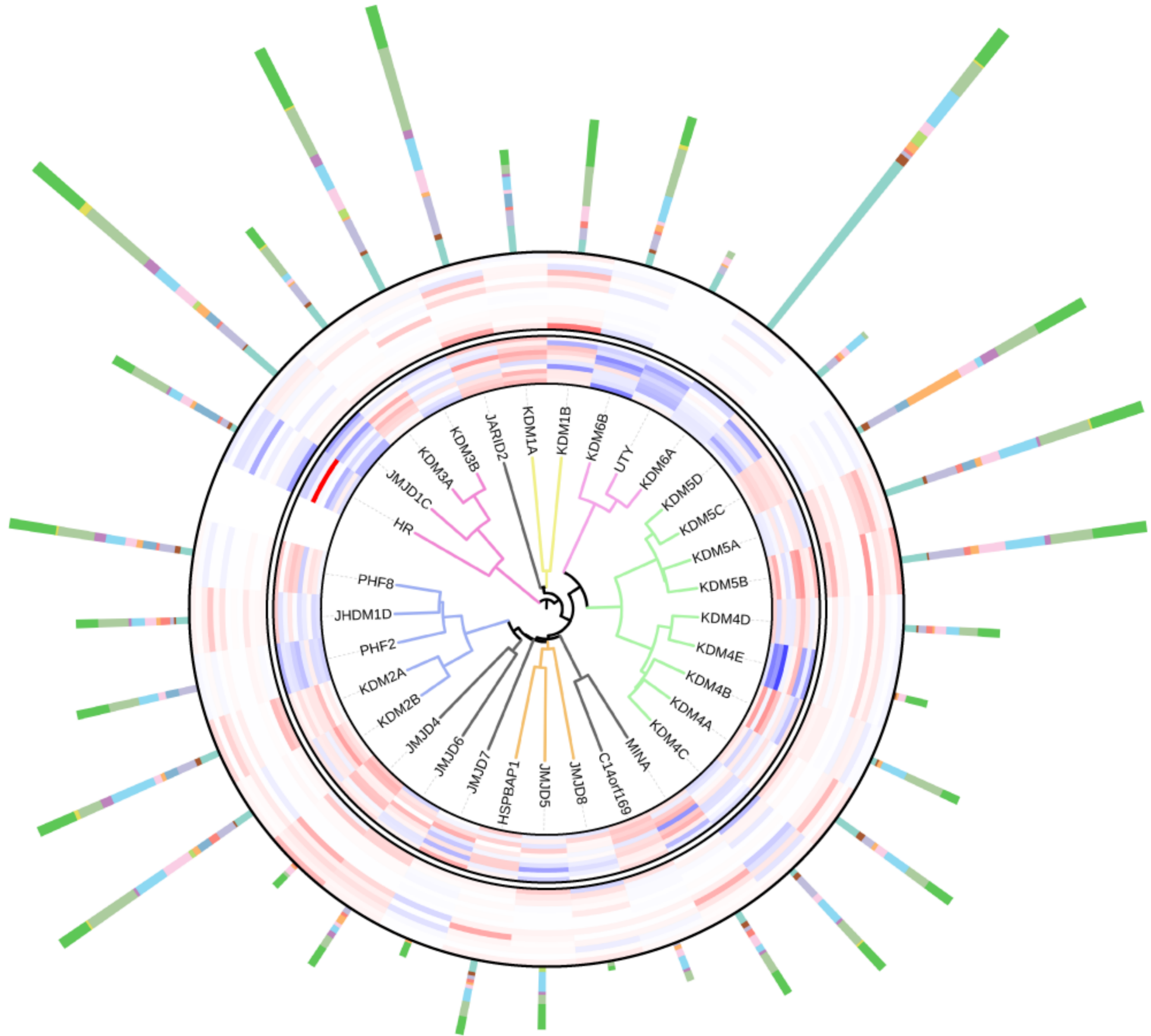



# SWI/SNF complex

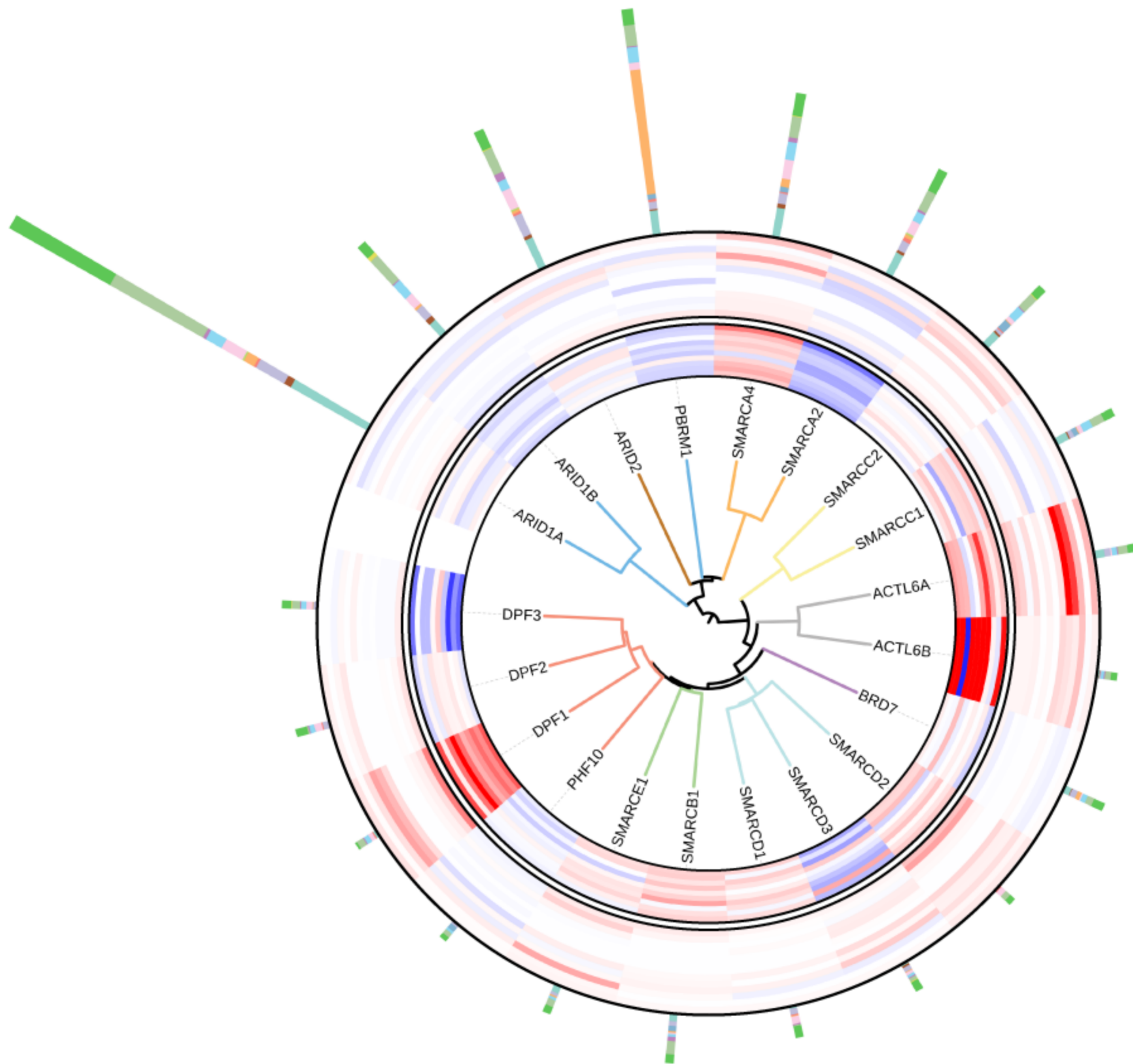

Supplement: Additional file 6 — Genomic alterations of HATs, HDACs, HMTs, HDMs and members of the SWI/SNF complex. The compositions of the plots are explained in Figure 1. [file 1471-2164-16-S8-S5-S6.pdf]

A)

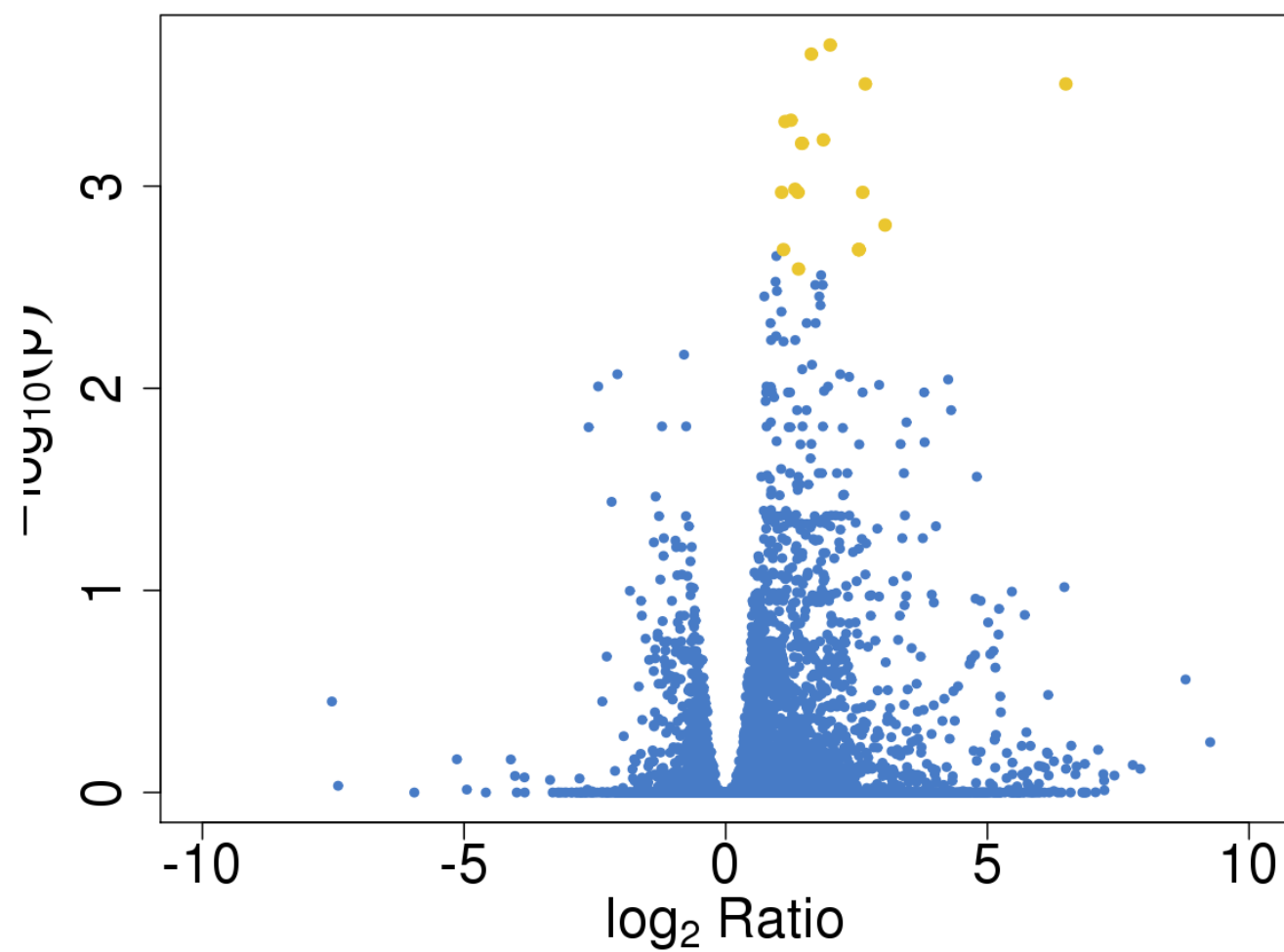

B)

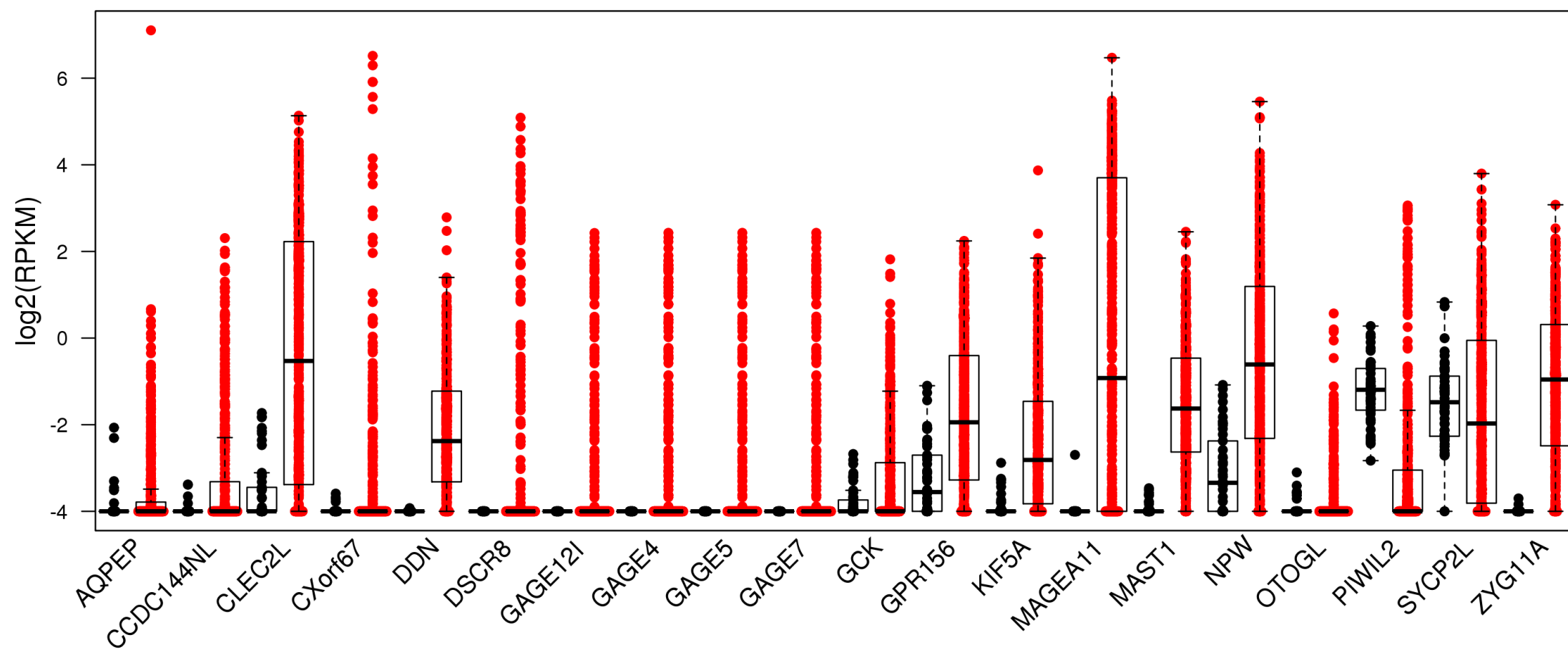

Supplement: Additional file 9 — Co-expression of cancer testis genes. A) Volcano plot resulting from the differential expression analysis between BRDT expressing and BRDT non-expressing LUSC tumors. B) Gene expression levels of co-expressed cancer testis in LUSC (black: healthy tissues, red: tumors). [file 1471-2164-16-S8-S5-S9.pdf]

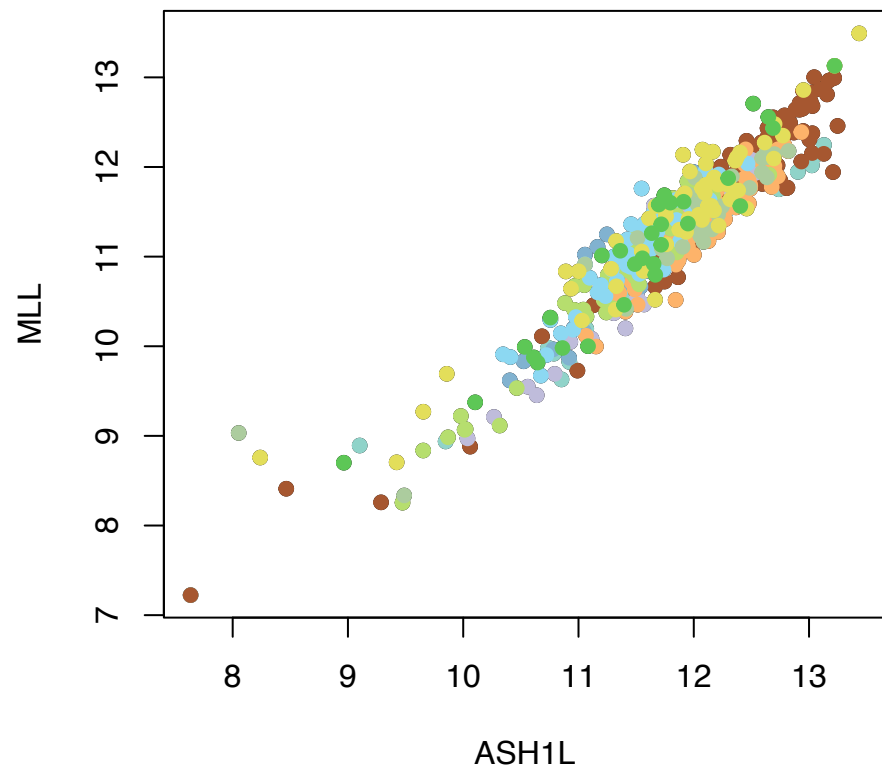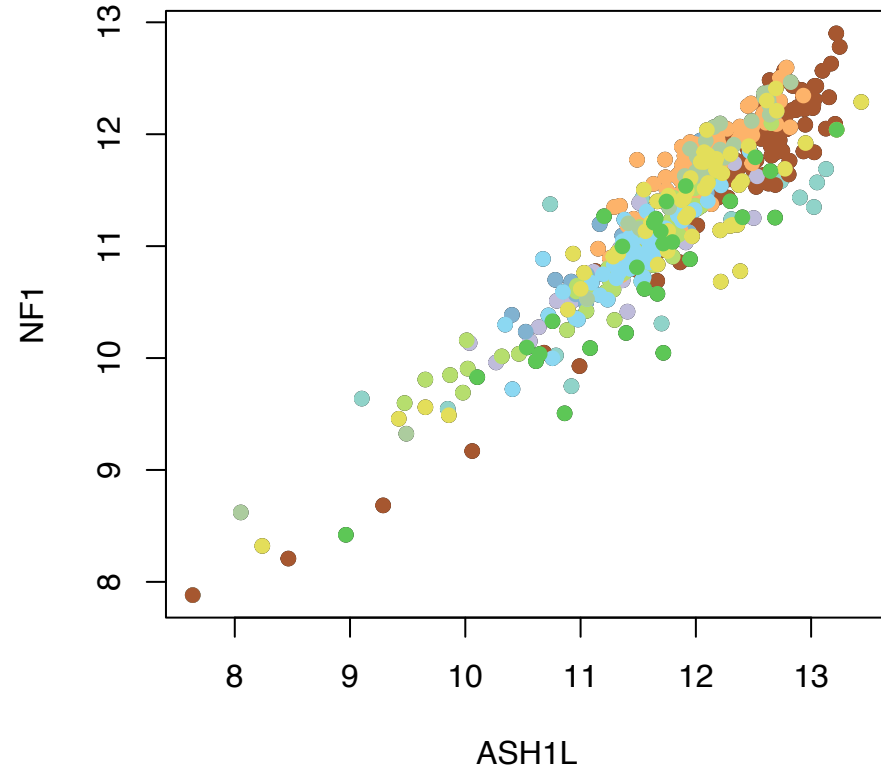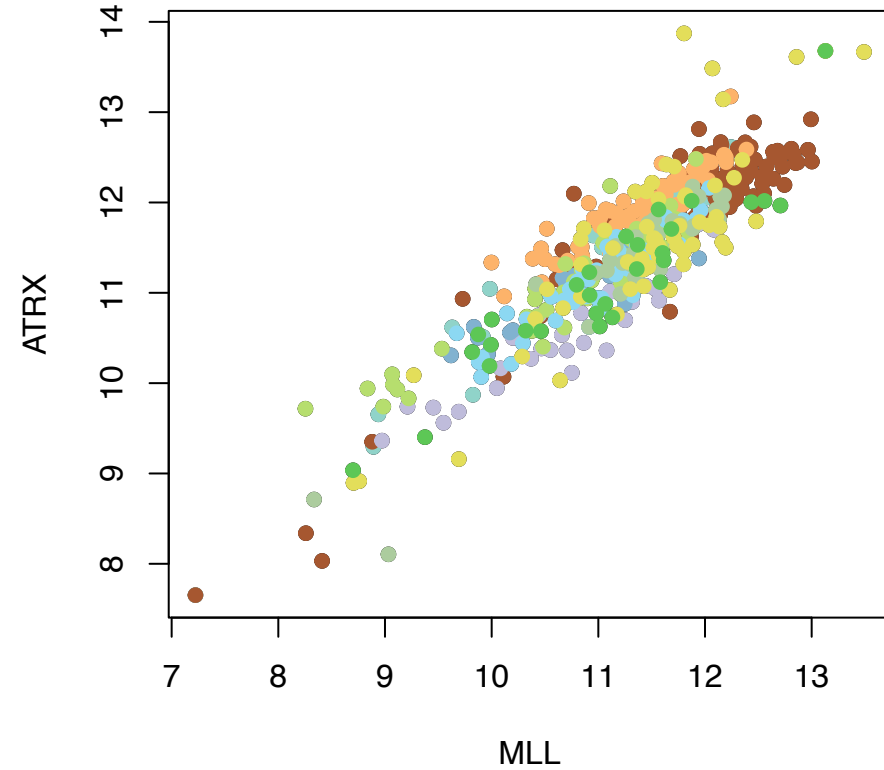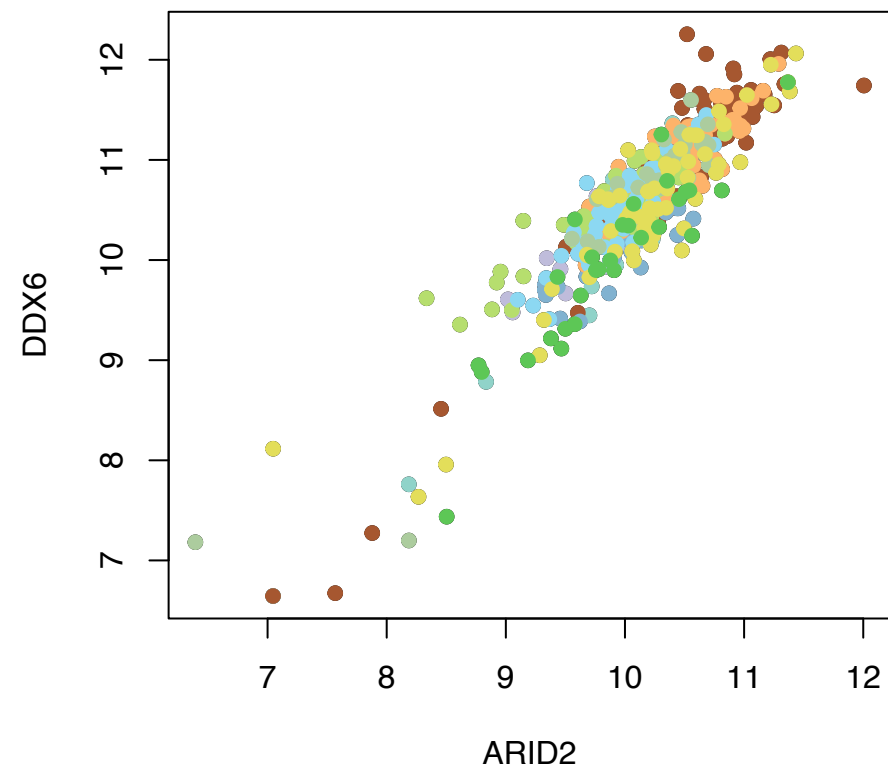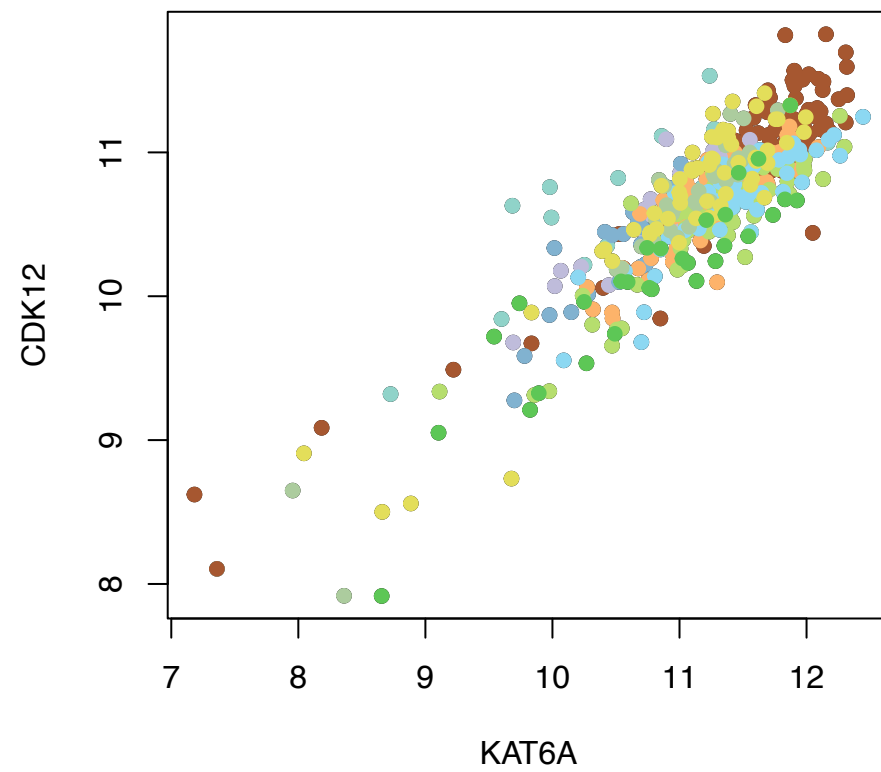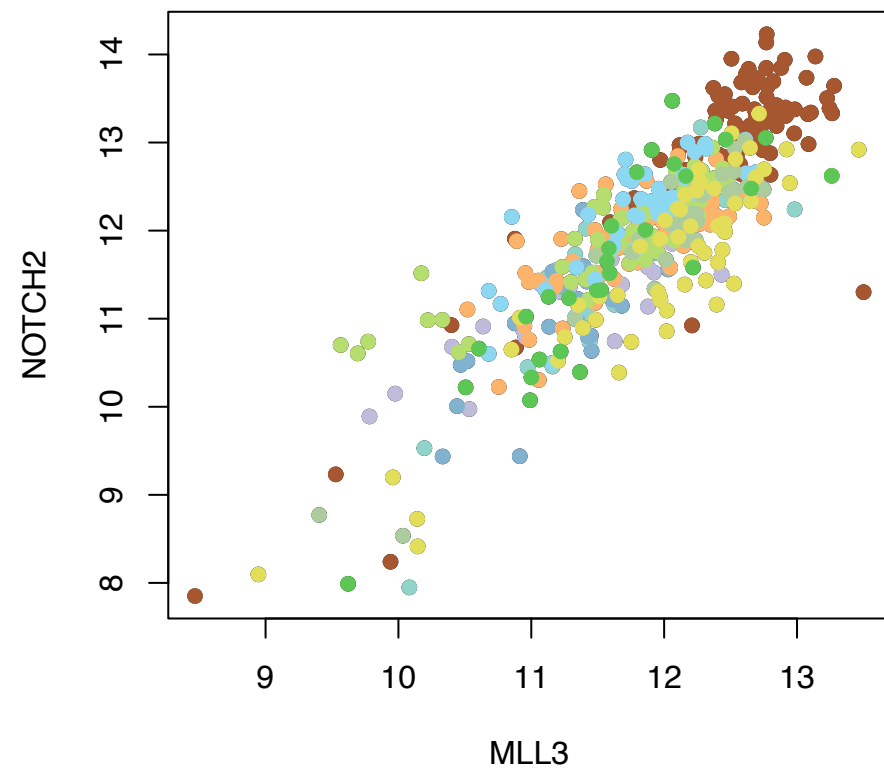

Supplement: Additional file 11 — Examples of co-expression between ERGs and other genes in healthy tissues. Each dot reflects the gene expression levels (represented by variance stabilized RNAseq count data) of the ERG (x axis) and the co-expressed gene (y axis). Dots are colored according to the associated tissue indication. [file 1471-2164-16-S8-S5-S11.pdf]

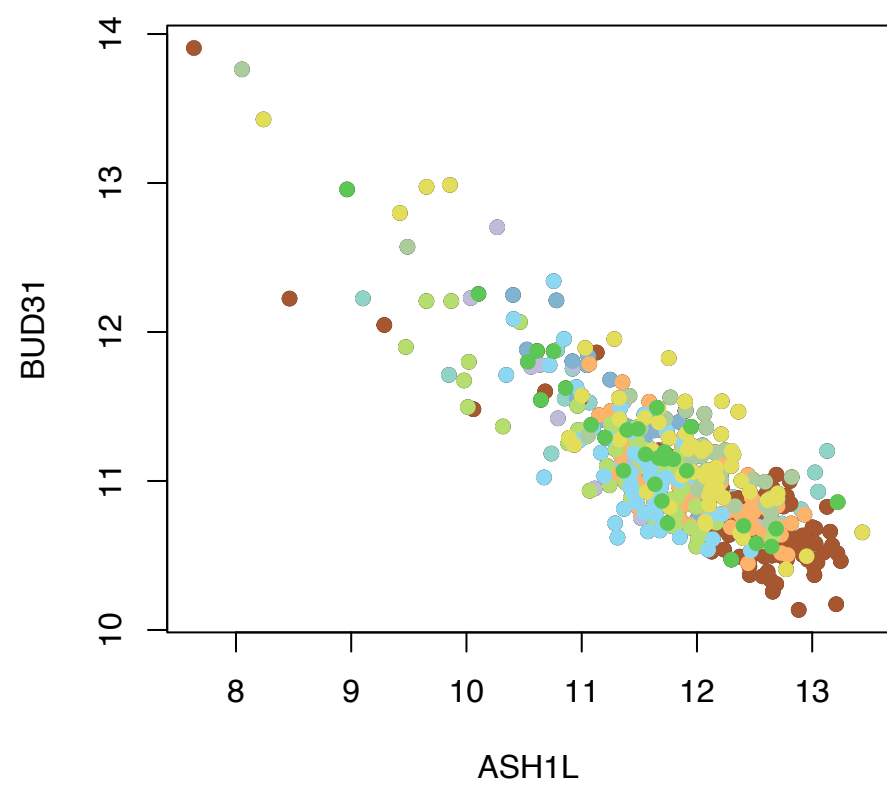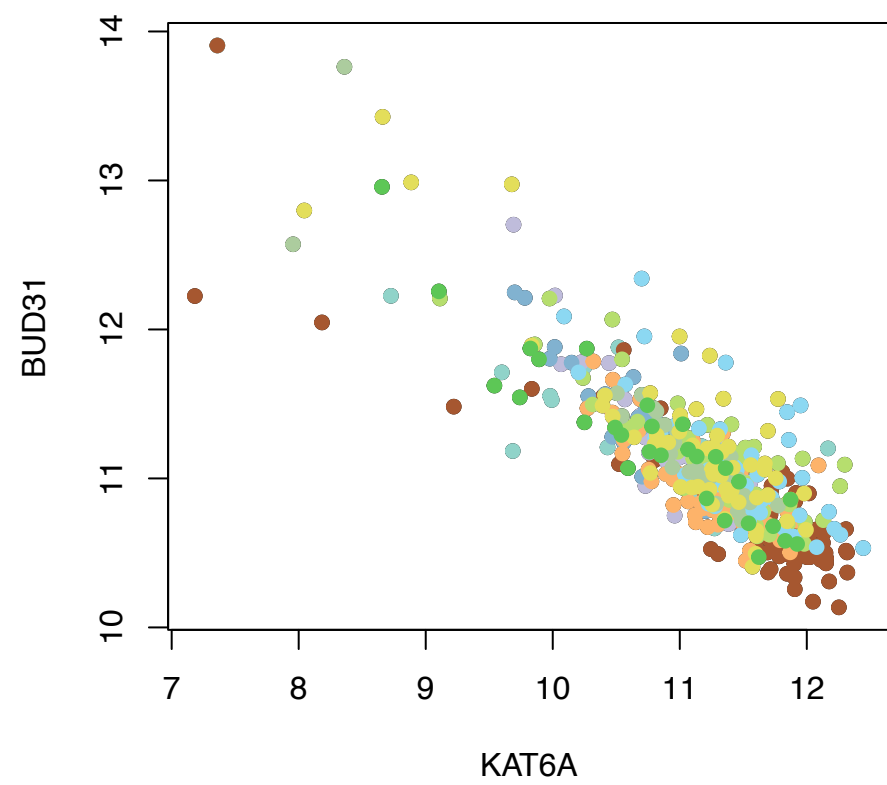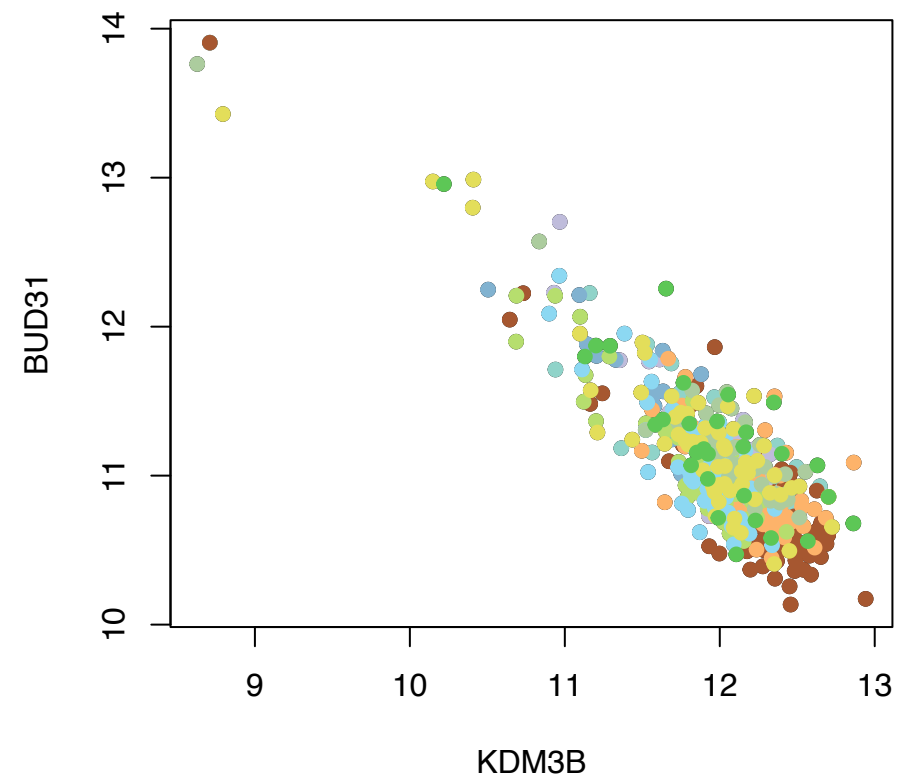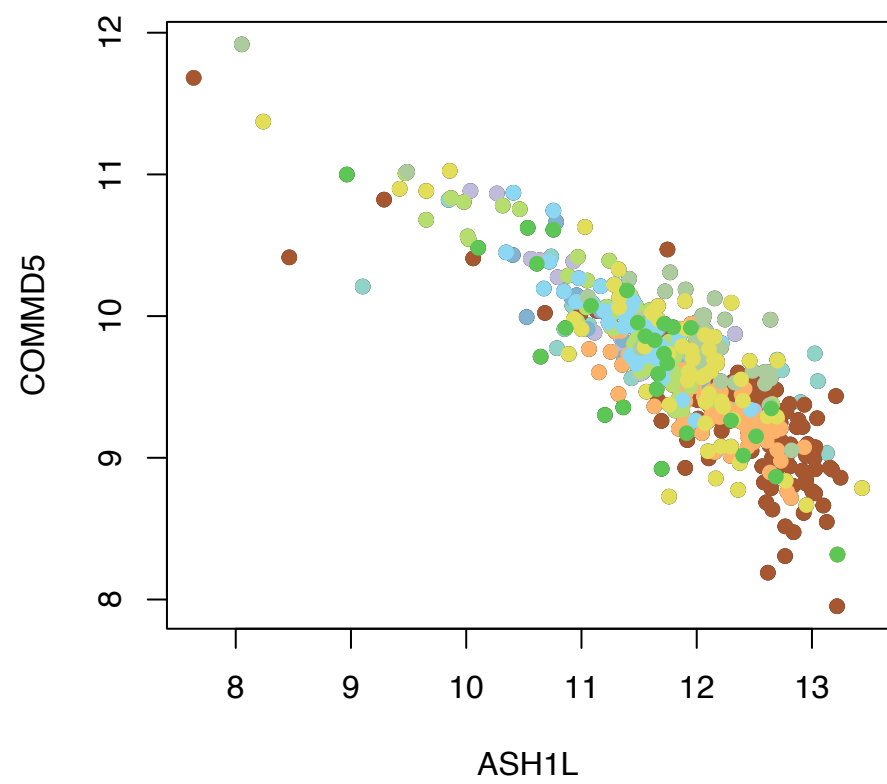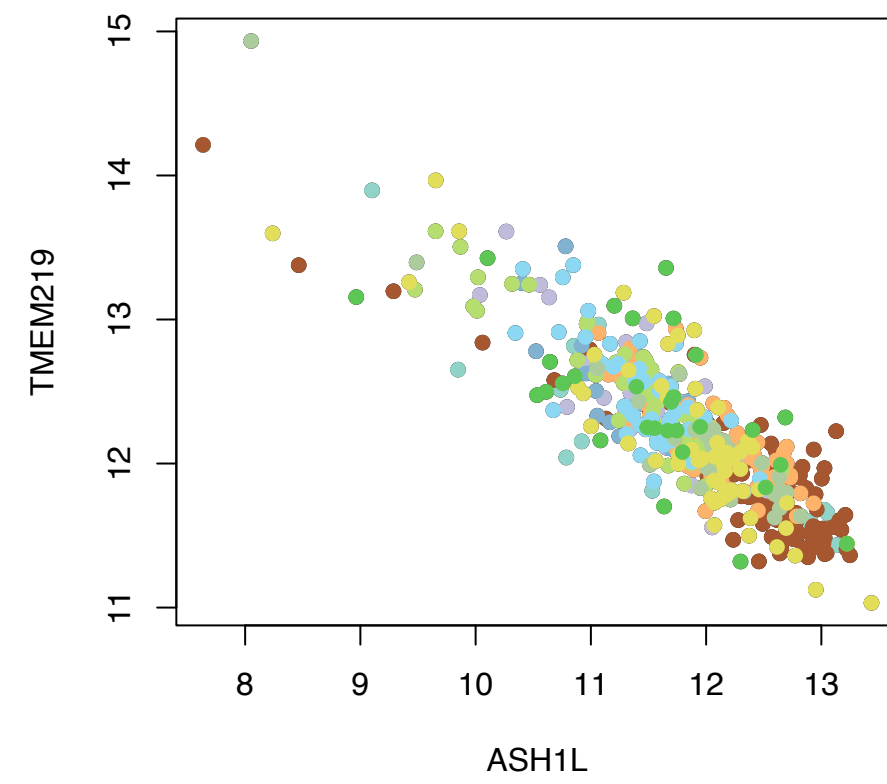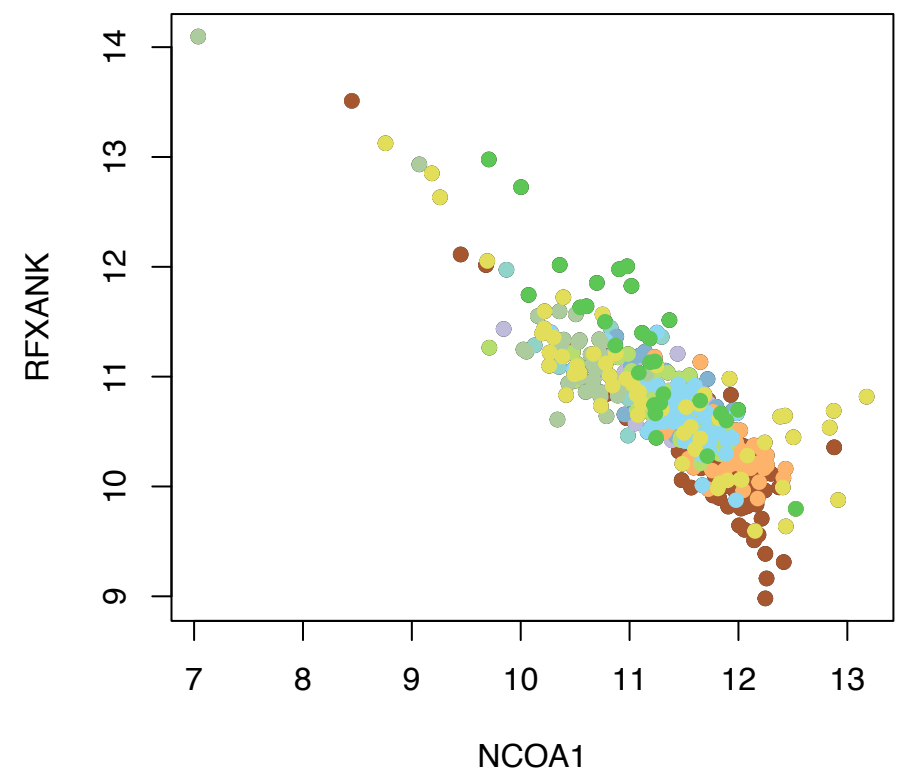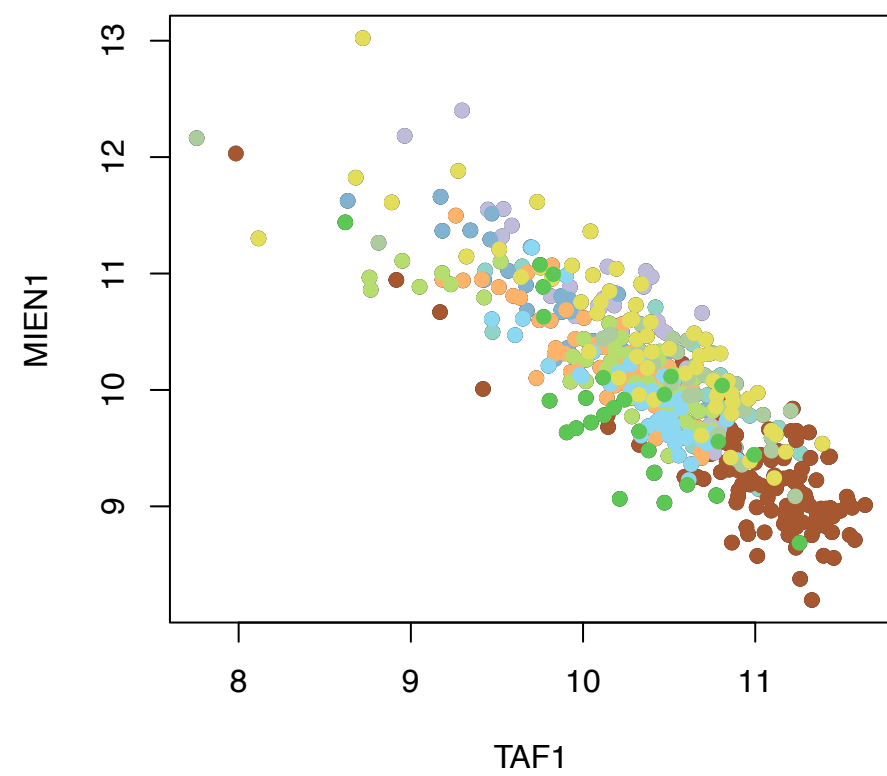

Supplement: Additional file 12 — Negative correlations between expression levels of ERGs and other genes. Analogously gene expression (variance stabilized RNAseq count data) of the epigenetic regulator (x axis) and the co-expressed gene (y axis). Colors indicate the associated tissue indication. [file 1471-2164-16-S8-S5-S12.pdf]

# Degree of H3K27me3

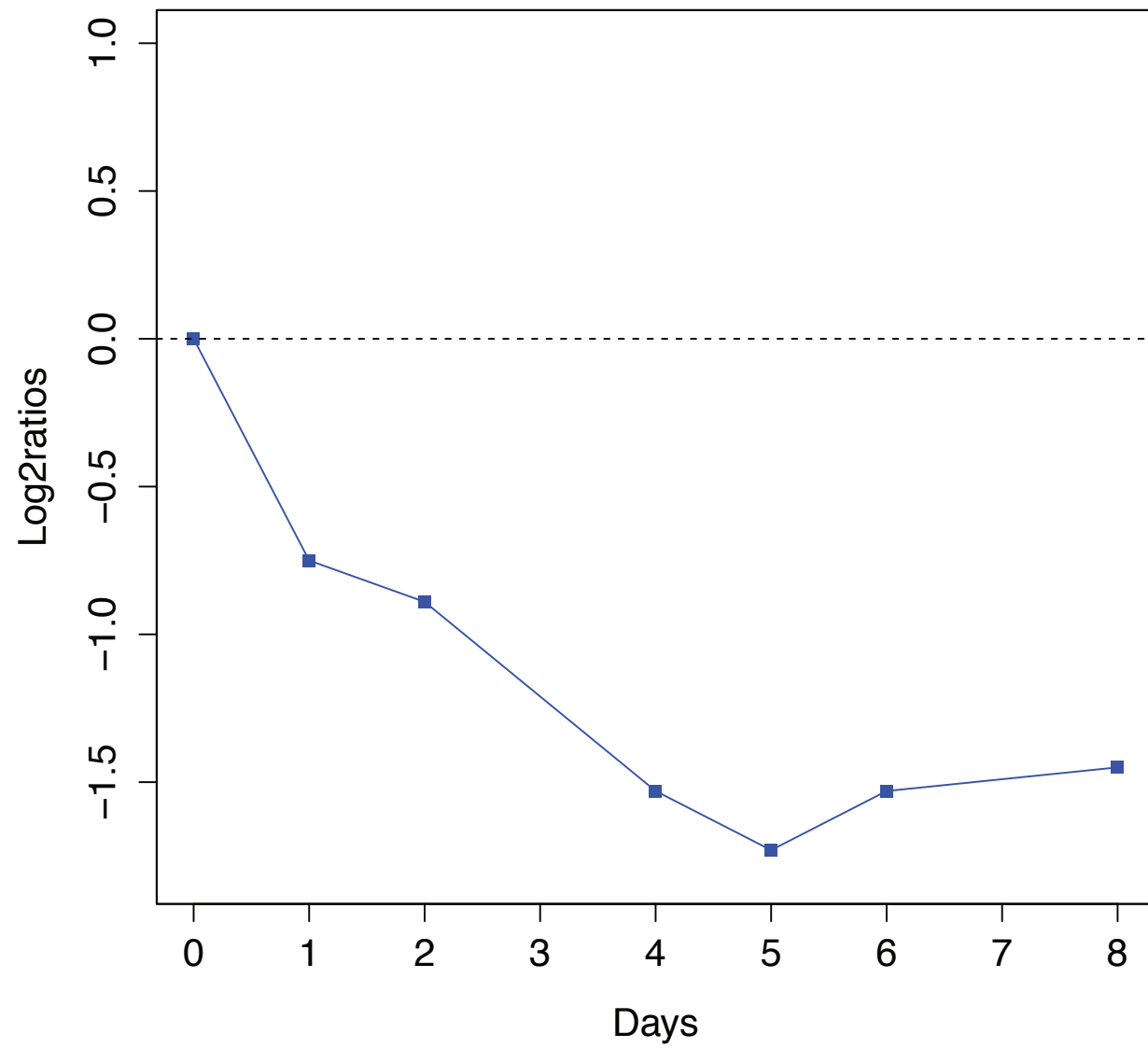

Supplement: Additional file 14 — Mass spectrometry based quantitation of H3K27me following EZH2 inhibition. [file 1471-2164-16-S8-S5-S14.pdf]
