# Supplementary material for: Bioinformatics analysis of thousands of TCGA tumors to determine the involvement of epigenetic regulators in human cancer
Source: BMC Genomics. 2015 Jun 18;16(Suppl 8):S5. doi: 10.1186/1471-2164-16-S8-S5 (PMC4480953; doi:10.1186/1471-2164-16-S8-S5)
Supplement: Additional file 2 — Description of genomic features. [file 1471-2164-16-S8-S5-S2.pdf]

Description of genomic features

Genomic features

Using TCGA data we employed 48 features associated with mutation, expression, or copy number alterations for each human gene:

Feature 1: Mutation selection score

The entropy as a measure of mutation recurrence within a gene were calculated as

$$S = \sum_{i=1}^{x} -f_i \cdot \ln(f_i)$$

where x is the number of different somatic non-synonymous mutations within a gene, f_i is the frequency of each mutation relative to the total number n of all mutations within a gene over all tumor samples, and ln is the natural logarithm.

The maximum entropy S_0 implies the maximum degree of randomness with each mutation having the same probability $p_i = 1/x$. S_0 is calculated as

$$S_0 = \sum_{i=1}^{x} -p_i \cdot \ln(p_i) = \ln(x)$$

The 'mutation selection score' reflects the difference between the maximum entropy S_0 and the given entropy S ($\Delta S = S_0 - S$). Tumor-specific hot spot mutations are selected during tumor evolution and therefore occur at high frequency in the tumor panel.

Features 2-29: Mutation frequency normalized by coding length

Mutation Assessor (http://mutationassessor.org) was used to distinguish between missense mutations with high (HiFI) or low (LoFI) functional impact. Loss of

function (LOF) mutations were defined as the combination of nonsense and frameshift mutations.

For each gene the proportions of tumors (F_t) with a minimum of one mutation of class m_c were determined. We defined non-synonymous, missense, HiFI, LoFI, splice site, LOF, or total mutations as different classes, and calculated m_c values for each mutation class separately. m_c values in a given cancer type t were calculated as

$$F_t = \frac{M_c}{N}$$

where N is the total number of tumors in cancer type t, and M_c is the total number of tumors with minimum one mutation of class m_c

To determine the mutation frequency (F_c) among all cancer types normalized by coding sequence, we calculated the following score for each mutation class:

$$F_c = (\sum_{BLCA}^{UCEC} F_t)/c^d$$

where c reflects the coding sequence length and d ranges from 1 to 4.

With 7 different mutation classes and d ranging from 1 to 4, the resulting 28 scores were used as genomic features for cancer gene prediction. Notably, to compare resulting F_c values between studies that are based on a different number of cancer types, F_c needs to be normalized by the number of included cancer types.

Features 30-44: Mutation frequency relative to low impact mutation or background mutation

Silent mutation or benign mutations as the combination of silent and LoFI were taken as a measure for the background mutation rate of each gene. For each gene, mutation frequencies (M_c) of different mutation classes c were normalized

by the background mutation rate or the number of LoFI mutations for each cancer type t:

$$p_t = (M_c^a + m_c^a)/(M_c^b + m_c^b)$$

M_c^a and M_c^b reflect the total number of tumors with minimum one mutation of classes a and b, respectively. m_c^a and m_c^b reflect the medians of M_c^a and M_c^b, respectively, of all human genes.

To determine the mutation frequencies among all cancer types (p_c), we calculated the sum of all cancer type specific mutation frequencies:

$$p_c = \sum_{BLCA}^{UCEC} p_t$$

We compared the following mutation classes resulting in 15 features:

- HiFI / LoFI mutations
- Splice site / LoFI mutations
- LOF / LoFI mutations

- HiFI / silent mutations
- LoFI / silent mutations
- Splice site / silent mutations
- LOF / silent mutations

- HiFI / benign mutations
- LoFI / benign mutations
- Splice site / benign mutations
- LOF / benign mutations

- HiFI / total mutations

- LoFI / total mutations
- Splice site / total mutations
- LOF / total mutations

Features 45-46: Differential gene expression scores

Log-scaled fold changes resulting from differential expression analysis between tumors and healthy tissues formed one empirical expression based feature:

$$E_c = \sum_{BLCA}^{UCEC} DE_c$$

DE_c reflects the medium fold change of the in a given cancer type.

As an alternative expression feature that takes the significance of the differential gene expression analysis into account, adjusted p values were used:

$$E_p = \sum_{BLCA}^{UCEC} r * DE_p$$

$DE_c > 0$: r = 1

$DE_c < 0$: r = -1

Features 47-48: Copy number features

Using GISTIC, the extent of copy number deletions and amplifications for each gene in each cancer study was determined as the proportion of tumors with ‘deep loss’ and ‘high-level gain’ changes, respectively.

The sums of all cancer type specific amplification or deletion frequencies formed the two copy number features.
