# Supplementary material for: Bioinformatics analysis of thousands of TCGA tumors to determine the involvement of epigenetic regulators in human cancer
Source: BMC Genomics. 2015 Jun 18;16(Suppl 8):S5. doi: 10.1186/1471-2164-16-S8-S5 (PMC4480953; doi:10.1186/1471-2164-16-S8-S5)
Supplement: Additional file 5 — Overview: Cancer gene prediction applied to ERGs. [file 1471-2164-16-S8-S5-S5.pdf]

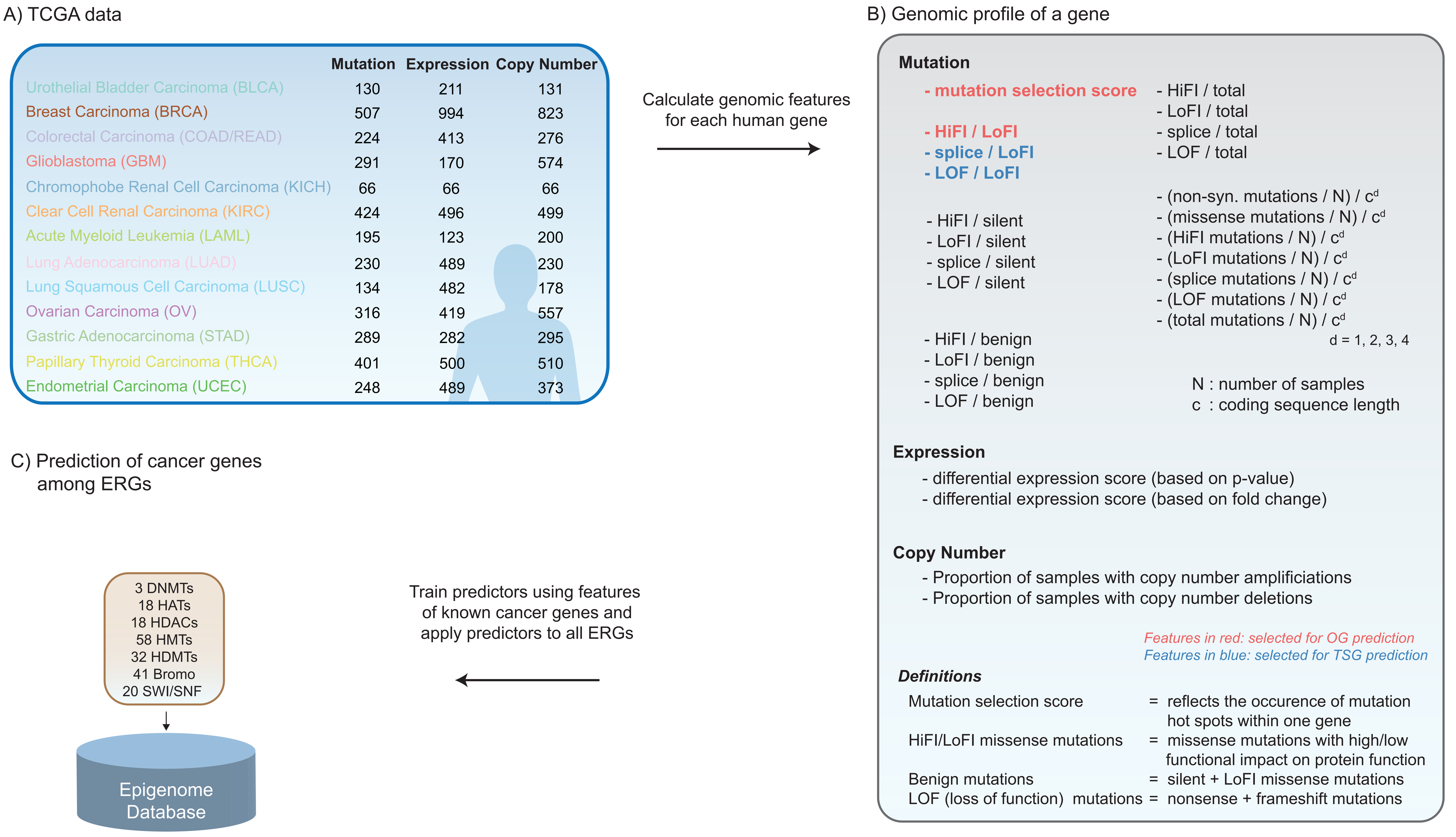
A) TCGA data

	Mutation	Expression	Copy Number
Urothelial Bladder Carcinoma (BLCA)	130	211	131
Breast Carcinoma (BRCA)	507	994	823
Colorectal Carcinoma (COAD/READ)	224	413	276
Glioblastoma (GBM)	291	170	574
Chromophobe Renal Cell Carcinoma (KICH)	66	66	66
Clear Cell Renal Carcinoma (KIRC)	424	496	499
Acute Myeloid Leukemia (LAML)	195	123	200
Lung Adenocarcinoma (LUAD)	230	489	230
Lung Squamous Cell Carcinoma (LUSC)	134	482	178
Ovarian Carcinoma (OV)	316	419	557
Gastric Adenocarcinoma (STAD)	289	282	295
Papillary Thyroid Carcinoma (THCA)	401	500	510
Endometrial Carcinoma (UCEC)	248	489	373
