# Supplementary material for: Bioinformatics analysis of thousands of TCGA tumors to determine the involvement of epigenetic regulators in human cancer
Source: BMC Genomics. 2015 Jun 18;16(Suppl 8):S5. doi: 10.1186/1471-2164-16-S8-S5 (PMC4480953; doi:10.1186/1471-2164-16-S8-S5)
Supplement: Additional file 13 — Known models for EZH2 as cell cycle regulator. Two established models describe a cell cycle regulating role of EZH2: With its transcription repressing role as member of PRC2 complex (left panel), EZH2 enhances the expression of cell cycle regulators indirectly by repressing associated tumor suppressors such as CDKN1C. In an alternative model, EZH2 acts as a direct activator (right panel). Phosphorylated EZH2 activates STAT3 via methylation, which in turn activates the cyclin D1/CDK2 complex. Interestingly CDK1 and CDK2 have been shown to phosphorylate EZH2. In addition EZH2 has been shown to inhibit BRCA1 phosphorylation presumably via interaction with Akt-1 resulting into increase of cell cycle promoting CDC25C. [file 1471-2164-16-S8-S5-S13.pdf]

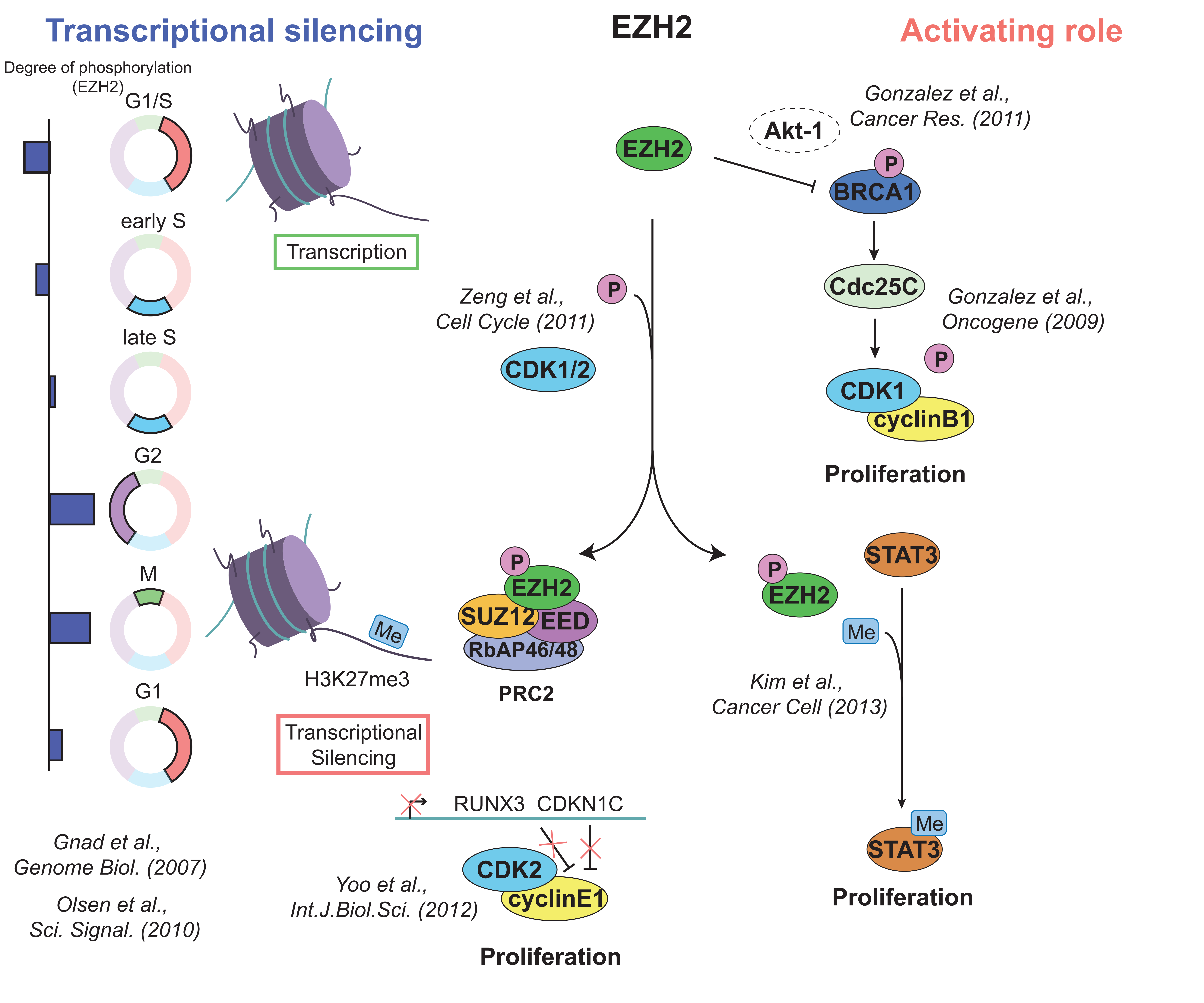

Transcriptional silencing
EZH2
Activating role
Degree of phosphorylation (EZH2)
G1/S
early S
late S
G2
M
G1
Transcription
H3K27me3
Transcriptional Silencing
Me
Gnad et al., Genome Biol. (2007)
Olsen et al., Sci. Signal. (2010)
EZH2
Zeng et al., Cell Cycle (2011)
P
CDK1/2
P
EZH2
SUZ12
EED
RbAP46/48
PRC2
RUNX3 CDKN1C
CDK2
cyclinE1
Yoo et al., Int.J.Biol.Sci. (2012)
Proliferation
Akt-1
Gonzalez et al., Cancer Res. (2011)
P
BRCA1
Cdc25C
Gonzalez et al., Oncogene (2009)
P
CDK1
cyclinB1
Proliferation
STAT3
P
EZH2
Me
Kim et al., Cancer Cell (2013)
Me
STAT3
Proliferation
