# Supplementary material for: Bioinformatics analysis of thousands of TCGA tumors to determine the involvement of epigenetic regulators in human cancer
Source: BMC Genomics. 2015 Jun 18;16(Suppl 8):S5. doi: 10.1186/1471-2164-16-S8-S5 (PMC4480953; doi:10.1186/1471-2164-16-S8-S5)
Supplement: Additional file 16 — Clustered time series. Using fuzzy c-means clustering, time course profiles formed three clusters of down-, up-, and non-regulated proteins. Colors reflect the similarities between specific time series and the associated cluster. [file 1471-2164-16-S8-S5-S16.pdf]

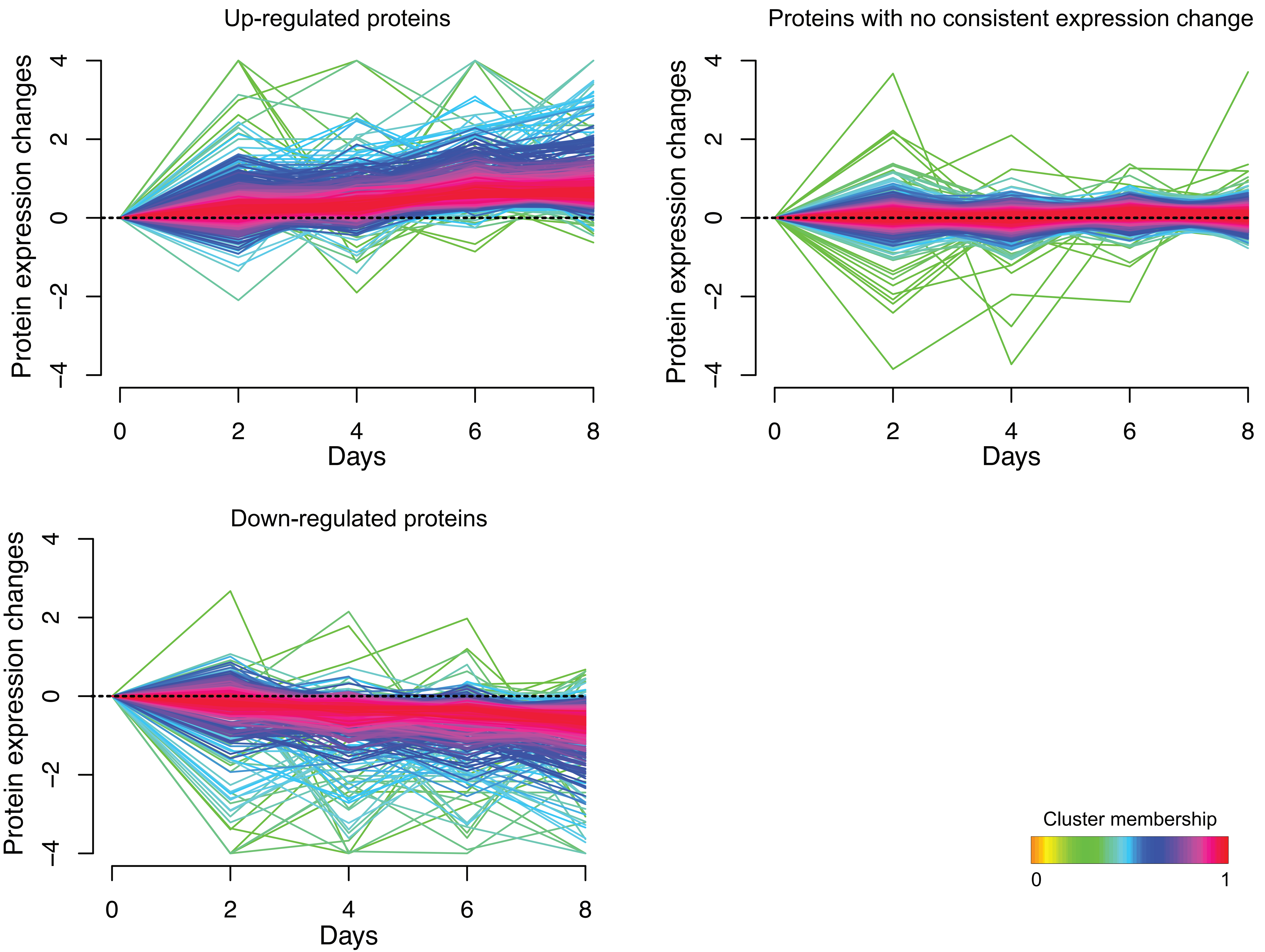
Up-regulated proteins
Proteins with no consistent expression change
Down-regulated proteins
Protein expression changes
Days
4
2
0
−2
−4
0
2
4
6
8
Cluster membership
0
1
